# Effects of probiotics and synbiotics on liver enzymes, glucose and lipid metabolism, and inflammatory markers in patients with metabolic dysfunction-associated steatotic liver disease: a systematic review and meta-analysis

**DOI:** 10.3389/fnut.2026.1940415

**Published:** 2026-08-25

**Authors:** Ying Jiang, Yaru Shi, Jianzhong Cao

**Affiliations:** School of Traditional Chinese Medicine, Hunan University of Chinese Medicine, Changsha, Hunan, China

**Keywords:** gut microbiota, meta-analysis, metabolic dysfunction-associated steatotic liver disease, probiotics, randomized controlled trial, synbiotics

## Abstract

**Objective:**

The potential of probiotics and synbiotics as a targeted microbiome intervention for metabolic dysfunction-associated steatotic liver disease (MASLD/NAFLD) has garnered significant attention; however, their clinical efficacy exhibits substantial heterogeneity. This study aimed to systematically evaluate the clinical efficacy of adjunctive probiotic and synbiotic therapy in MASLD/NAFLD and explore the sources of this heterogeneity.

**Methods:**

A systematic search was conducted across PubMed, Web of Science, Embase, and The Cochrane Library for randomized controlled trials (RCTs) published up to June 2026. RevMan 5.3 and Stata 17.0 were utilized to pool effect sizes [mean difference (MD)/standardized mean difference (SMD)]. Subgroup analyses and the GRADE system were employed to investigate heterogeneity and assess the quality of evidence.

**Results:**

A total of 30 RCT publications encompassing 1,516 unique patients were included. The meta-analysis demonstrated that probiotics and synbiotics significantly mitigated liver injury (ALT, AST) and systemic inflammation (TNF-α), while improving specific metabolic parameters (FPG, TC, HDL-C) (*p* < 0.05). Although the overall effects on triglycerides (TG) and insulin resistance (HOMA-IR) were not statistically significant, subgroup analyses revealed more pronounced benefits in glucolipid metabolism among younger populations (<50 years), Asian cohorts, and those receiving short-to-medium-term interventions (<24 weeks). Furthermore, insulin resistance was significantly ameliorated in non-diabetic MASLD/NAFLD patients.

**Conclusion:**

As an adjunct to lifestyle interventions, probiotics and synbiotics effectively ameliorate liver injury and systemic inflammation in patients with MASLD/NAFLD. Their regulatory efficacy on lipid metabolism and insulin resistance is significantly influenced by variables including patient age, geographical region, and intervention duration. For younger, non-diabetic MASLD/NAFLD patients, short-to-medium-term probiotic interventions (<24 weeks) yield optimal metabolic benefits. These findings underscore the necessity of adopting precision microbiome-targeted intervention strategies based on patient stratification in future clinical practice.

**Systematic review registration:**

https://www.crd.york.ac.uk/PROSPERO/view/CRD420261442665, identifier PROSPERO (CRD420261442665).

## 1 Introduction

Non-alcoholic fatty liver disease (NAFLD), now reclassified as metabolic dysfunction-associated steatotic liver disease (MASLD), has emerged as one of the leading causes of chronic liver disease and liver-related mortality worldwide. This condition currently affects over 32% of adults and 7 to 14% of children globally (1, 2). With incidence rates continuing to climb rapidly, its global prevalence is projected to surpass 55.4% by 2040 (3). Epidemiological data exhibit significant regional heterogeneity, with the highest prevalence observed in Latin America (44.4%) and the lowest in Western Europe (25.1%) (3). The disease spectrum encompasses simple steatosis, metabolic dysfunction-associated steatohepatitis (MASH, formerly NASH), hepatic fibrosis, and cirrhosis, ultimately progressing to hepatocellular carcinoma (HCC) (4). Beyond contributing to substantial liver-related mortality, MASLD/NAFLD is inextricably linked to type 2 diabetes mellitus (T2DM) and metabolic syndrome (5). Notably, patients with T2DM have an 11-fold higher risk of developing concurrent MASLD/NAFLD compared to metabolically healthy individuals (6). According to the Global Burden of Disease study, MASLD/NAFLD accounts for approximately 170,000 cases of liver cancer, representing roughly 26% of all liver cancer cases worldwide (7, 8). Despite the substantial and widespread global prevalence, only 32 countries have formulated specific clinical guidelines, indicating the absence of a comprehensive public health response mechanism (9). Faced with its high prevalence, substantial mortality rate, and heavy economic burden (10), therapeutic options for MASLD remain limited. Resmetirom, a thyroid hormone receptor-*β* agonist, became the first drug approved by the U.S. Food and Drug Administration (FDA) in March 2024 for the treatment of adults with non-cirrhotic MASH and moderate-to-advanced liver fibrosis (consistent with stages F2 to F3), to be used in conjunction with diet and exercise (11, 12). However, its indication is restricted to this specific subgroup, and the treatment needs of the broader MASLD population remain largely unmet. Consequently, clinical management continues to rely primarily on lifestyle interventions, such as dietary modification, weight reduction, and physical exercise (13).

In recent years, the “gut-liver axis” theory has provided a novel perspective for elucidating the pathological mechanisms of MASLD/NAFLD. When gut barrier function is impaired or dysbiosis occurs, intestinal mucosal permeability increases, facilitating the massive translocation of microbial components and metabolites [such as the endotoxin lipopolysaccharide (LPS)] from the intestinal lumen into the bloodstream, reaching the liver directly via the portal vein (14). These pathogen-associated molecular patterns (PAMPs) can activate signaling pathways such as Toll-like receptor 4 (TLR4) in the liver, thereby driving the progression of hepatic steatosis, inflammation, and fibrosis (15). Concurrently, the gut microbiota structure in MASLD/NAFLD patients often exhibits characteristic dysbiosis, primarily manifesting as an imbalance in the ratio of Bacteroidetes to Firmicutes and an increased abundance of endotoxin-producing Gram-negative bacteria. This microecological profile is highly correlated with intrahepatic lipid deposition and systemic inflammation (16).

Intervention strategies targeting the “gut-liver axis” have emerged as a research hotspot in the treatment of MASLD/NAFLD. Probiotics are defined as “live microorganisms that, when administered in adequate amounts, confer a health benefit on the host” (17). Their potential hepatoprotective mechanisms are characterized by multi-target effects. First, probiotics can competitively inhibit pathogenic bacteria and upregulate the expression of tight junction proteins to reinforce the integrity of the intestinal barrier, thereby effectively reducing the translocation of bacterial endotoxins (e.g., LPS) and other harmful molecules into the portal vein and curbing endotoxemia (18). Second, as the load of LPS entering the liver decreases, the overactivation of TLR4 on the surfaces of Kupffer cells and hepatic stellate cells is suppressed. This subsequently blocks downstream pro-inflammatory signaling cascades, such as NF-κB, thereby alleviating hepatic inflammation and fibrosis (19, 20). Furthermore, metabolites secreted by probiotics, such as short-chain fatty acids (SCFAs), not only serve as energy substrates for intestinal epithelial cells to fortify the barrier but also enter the liver via the portal vein. Through the regulation of metabolic pathways such as AMPK, these metabolites ameliorate lipid accumulation and insulin resistance, ultimately restoring hepatic glucolipid metabolic homeostasis (21).

Therefore, targeted modulation of the gut microbiota possesses strong biological plausibility and clinical potential. However, although multiple randomized controlled trials (RCTs) have evaluated the clinical efficacy of probiotics, these studies exhibit high heterogeneity in terms of strain specificity, dosage, intervention duration, baseline subject characteristics, and outcome measures. Consequently, the generalizability of any single study is severely limited. Moreover, previous meta-analyses have not promptly incorporated the high-quality evidence that has emerged in recent years. In light of this, the present study comprehensively searched and systematically integrated the latest global clinical data. Employing a meta-analytical approach, we aimed to rigorously evaluate the ameliorative effects of probiotics and synbiotics as an adjunctive therapy on hepatic, metabolic, and systemic inflammatory parameters in patients with MASLD/NAFLD. The findings of this study are expected to provide more precise and reliable evidence-based medical guidance for clinical practice.

## 2 Methods

This study was designed, conducted, and reported in strict accordance with the Preferred Reporting Items for Systematic Reviews and Meta-Analyses (PRISMA) 2020 statement (22). The protocol for this systematic review has been registered in the PROSPERO database (Registration ID: CRD420261442665).

### 2.1 Inclusion and exclusion criteria

The inclusion criteria were established based on the PICOS framework: (1) Participants (P): Patients aged ≥ 18 years with a confirmed diagnosis of MASLD/NAFLD via liver imaging [e.g., ultrasound, computed tomography (CT), magnetic resonance imaging (MRI), liver elastography] or histopathological biopsy. There were no restrictions regarding sex, ethnicity, or disease duration. (2) Interventions (I): The experimental group received probiotic preparations (encompassing either single-strain or multi-strain formulations) of any dosage or strain composition, administered as an adjunct to lifestyle interventions (dietary modification/exercise) or routine standard of care, as well as synbiotics in which probiotics constituted the core active component, and multi-ingredient preparations containing probiotics supplemented with micronutrients (e.g., vitamins) at nutritional doses. (3) Comparisons (C): The control group received an equivalent placebo in combination with the same routine standard of care or lifestyle interventions as the experimental group. (4) Outcomes (O): Studies were required to provide valid quantitative data for at least one of the following categories of parameters: (1) Hepatic function parameters: Alanine aminotransferase (ALT), aspartate aminotransferase (AST), and gamma-glutamyl transferase (GGT). (2) Lipid metabolism parameters: Total cholesterol (TC), triglycerides (TG), low-density lipoprotein cholesterol (LDL-C), and high-density lipoprotein cholesterol (HDL-C). (3) Glucose metabolism parameters: Fasting plasma glucose (FPG), insulin, glycosylated hemoglobin (HbA1c), and homeostasis model assessment of insulin resistance (HOMA-IR). (4) Inflammatory markers: High-sensitivity C-reactive protein (hs-CRP), tumor necrosis factor-alpha (TNF-α), and interleukin-6 (IL-6). (5) Study Design (S): Randomized controlled trials (RCTs).

The exclusion criteria were as follows: (1) Hepatic steatosis attributable to other etiologies [e.g., alcoholic liver disease, viral hepatitis, hereditary metabolic disorders such as Wilson’s disease, drug-induced liver injury (DILI), and autoimmune liver diseases], or concurrent cirrhosis, hepatocellular carcinoma (HCC), or a history of liver transplantation. When multiple companion publications reported on the same clinical trial, they were treated as a single study, with different outcome measures extracted from each report to ensure that the same patient cohort contributed data only once to any single-outcome meta-analysis; (2) Duplicate publications, studies from which valid data could not be extracted, or those containing apparent data errors or inconsistencies; (3) Non-RCT study designs, including reviews, case reports, conference abstracts, animal experiments, and *in vitro* studies.

### 2.2 Literature search strategy

A systematic literature search was conducted across four electronic databases: PubMed, Web of Science, Embase, and The Cochrane Library, from their respective inceptions up to June 9, 2026. The search strategy utilized a combination of controlled vocabulary [e.g., Medical Subject Headings (MeSH) and Emtree] and free-text terms (searched within titles and abstracts). The search equations were tailored to the specific syntactical rules and characteristics of each database, with the detailed search strategies provided in Supplementary Table S1. The specific comprehensive search string was constructed as follows: (((Non alcoholic Fatty Liver Disease OR Nonalcoholic Fatty Liver OR Nonalcoholic Fatty Livers OR NAFLD OR Nonalcoholic Fatty Liver Disease OR Nonalcoholic Steatohepatitis OR Nonalcoholic Steatohepatitides OR Metabolic associated fatty liver disease OR MAFLD OR Metabolic dysfunction-associated steatotic liver disease OR MASLD OR Metabolic dysfunction associated fatty liver disease OR Fatty Liver OR Steatohepatitis OR Steatohepatitides OR Steatosis of Liver OR Visceral Steatosis OR Visceral Steatoses OR Liver Steatosis OR Liver Steatoses)) AND (Probiotic OR Probiotics OR Synbiotic OR Synbiotics OR Bifidobacterium OR Bifidobacteria OR Bacillus bifida OR Yeast OR *Saccharomyces cerevisiae* OR Saccharomyces italicus OR accharomyces oviformis OR S cerevisiae OR *S. cerevisiae* OR Saccharomyces uvarum var. melibiosus OR *Candida robusta* OR Saccharomyces capensis OR *Lactobacillus acidophilus* OR *Lactobacillus amylovorus* OR Lactobacill OR Lactic acid bacteria OR *Clostridium butyricum* OR Bacillus OR Natto Bacteria OR Streptococcus thermophiles OR Enterococcus)) AND (randomized controlled trial OR controlled clinical trial OR randomized).

### 2.3 Literature selection and data extraction

All retrieved records were imported into EndNote X9 reference management software, where duplicates were eliminated utilizing a combination of the software’s automated deduplication function and manual screening. Two independent researchers (Ying Jiang and Yaru Shi) conducted the primary screening by evaluating the titles and abstracts to exclude clearly irrelevant studies. Subsequently, the full texts of potentially eligible articles were retrieved and subjected to a secondary screening strictly in accordance with the predefined inclusion and exclusion criteria to determine the final included studies. The entire screening process was cross-checked by the two researchers; any discrepancies were resolved through discussion and consensus, or by consulting a third independent researcher for arbitration.

Data extraction was performed independently by two researchers using a predefined, standardized Excel spreadsheet to capture the following information: (1) Basic characteristics: first author, publication year, and country of origin; (2) Baseline data: sample size, sex ratio, age, body mass index (BMI), and body weight; (3) Intervention details: probiotic strain composition, single- or multi-strain formulation, daily dosage (in colony-forming units [CFU]), and intervention duration; (4) Control group regimen; (5) Outcome measures: post-intervention means and standard deviations (mean ± SD). If an original study reported only standard errors (SE), these were converted to standard deviations using the formula recommended by the Cochrane Handbook (23). In cases of incomplete or missing data, attempts were made to contact the corresponding authors via email to acquire the necessary information.

### 2.4 Risk of bias assessment of included studies

The methodological quality of the included studies was evaluated using the Cochrane Risk of Bias tool (23). The assessment encompassed the following domains: random sequence generation, allocation concealment, blinding of participants and personnel, incomplete outcome data, selective reporting bias, and other potential sources of bias. Each domain was independently categorized and judged as having a “low risk,” “high risk,” or “unclear risk” of bias. This assessment process was conducted independently by two researchers and subsequently cross-checked to ensure accuracy.

### 2.5 Statistical analysis

Data synthesis and meta-analysis were primarily conducted using Review Manager (RevMan) software, version 5.3 (The Cochrane Collaboration). For dichotomous outcomes, data were expressed as risk ratios (RRs) with their corresponding 95% confidence intervals (CIs). For continuous outcomes, the mean difference (MD) with its 95% CI was utilized as the effect size if the measurement units were consistent across studies; conversely, the standardized mean difference (SMD) with its 95% CI was applied when different units or measurement methods were employed. All effect sizes were presented with point estimates and 95% CIs. A *p*-value < 0.05 was considered to indicate statistical significance.

Statistical heterogeneity across the included studies was quantitatively evaluated using Cochran’s *Q* test and the *I*^2^ statistic. A fixed-effects model was employed for data synthesis if *p* ≥ 0.10 and *I*^2^ ≤ 50%, indicating non-significant or acceptable heterogeneity. In contrast, if *p* < 0.10 or *I*^2^ > 50%, suggesting substantial heterogeneity among the studies, a random-effects model was adopted. For outcomes exhibiting high heterogeneity, subgroup analyses and leave-one-out sensitivity analyses were conducted to explore the potential sources of this heterogeneity. Furthermore, Stata software, version 17.0 (StataCorp, College Station, TX, USA), was utilized to generate funnel plots and perform Egger’s linear regression test to assess for potential publication bias.

## 3 Results

### 3.1 Literature selection process and results

**Figure 1:**
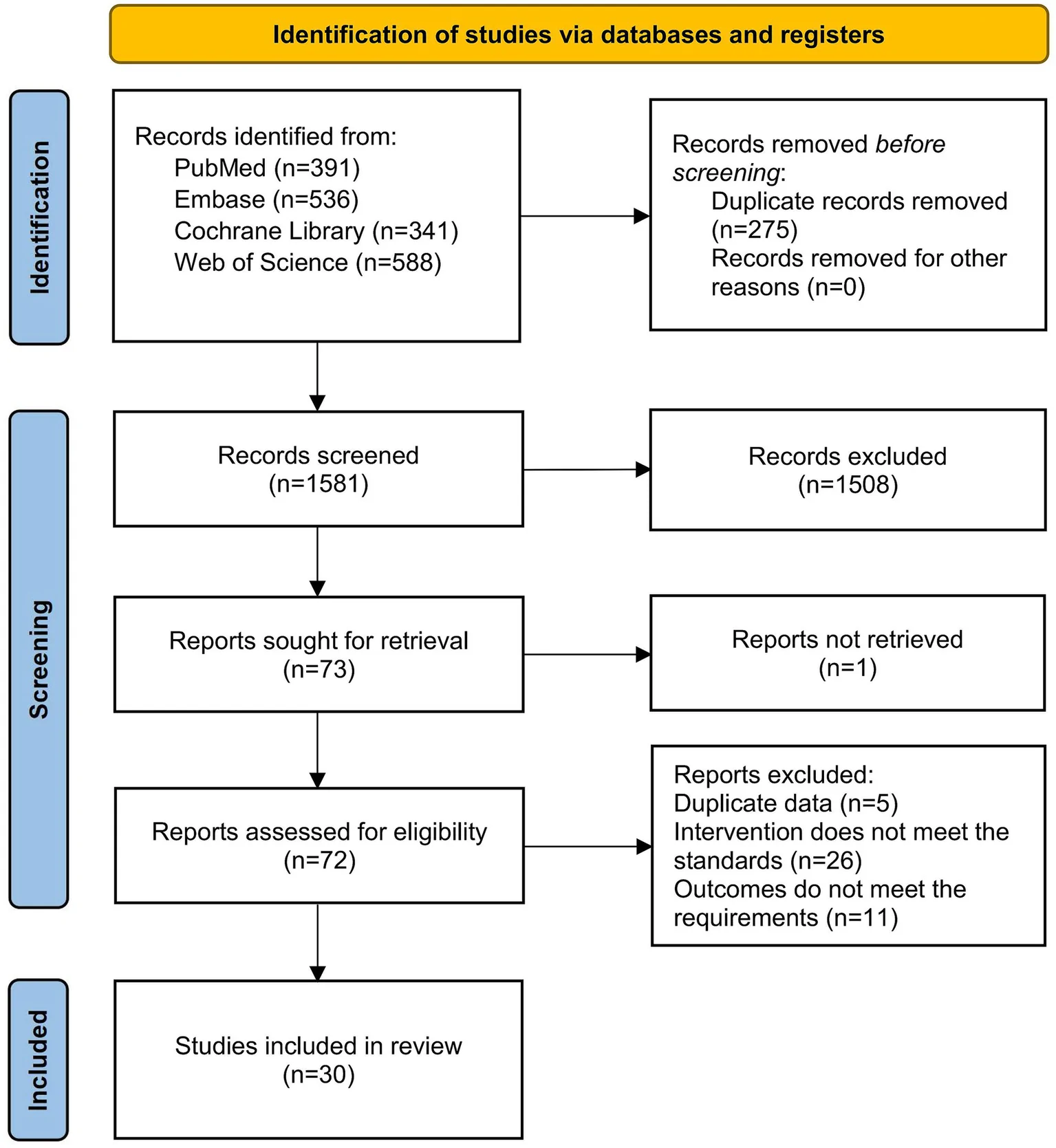
PRISMA flow diagram of the literature search and study selection process. This figure illustrates the flow of information through the different phases of a systematic review. Records were identified from PubMed, Embase, Cochrane Library, and Web of Science databases. After removing duplicates and screening by title/abstract and full text, 30 randomized controlled trials were finally included in the meta-analysis.

The initial systematic search across all electronic databases yielded a total of 1,856 articles. After removing duplicates utilizing EndNote X9 software, 1,581 articles remained. Following the primary screening of titles and abstracts, 1,509 irrelevant records (including animal experiments, reviews, conference abstracts, and non-RCTs) were excluded. The full texts of the remaining 72 articles were further evaluated. Of these, 42 articles were excluded for the following primary reasons: absence of a placebo or blank control, inappropriate outcome measures or lack of usable data, duplicate publications, and non-MASLD/NAFLD populations. Ultimately, a total of 30 RCTs (24–53) met the inclusion criteria and were incorporated into this meta-analysis. The detailed literature selection process is illustrated in Figure 1.

### 3.2 Baseline characteristics of included studies

This study ultimately included 30 eligible trials (24–53). The baseline characteristics of all included studies are summarized in detail in Table 1. Overall, the characteristics of the included studies presented the following distribution: The publication years of these 30 studies spanned from 2011 to 2026. The research was conducted across a wide geographical distribution, with the highest concentration of studies performed in Iran. The remaining studies originated from countries including Brazil, China, Malaysia, the United Kingdom, Italy, Spain, India, Ukraine, Serbia, and South Korea.

**Table 1:** Basic characteristics of included studies.

Study	Source	Sample	Female (%)	Age (years)	Intervention	Comparison	Treatment duration (weeks)
Aller et al. (25)	Spain	14/14	28.6	49.4	5 × 10^8^ CFU/d *Lactobacillus bulgaricus*, *Streptococcus thermophilus*	Placebo	12
Abhari et al. (24)	Iran	23/22	39.0	47.7	10^9^ CFU/d *B. coagulans* (GBI-30) plus	Placebo	12
Asgharian et al. (27)	Iran	38/36	82.5	46.6	*Lactobacillus casei*, Lactobacillus acidophilu, *Lactobacillus rhamnosus*, *Lactobacillus bulgaricus*, *Bifidobacterium breve*, *Bifidobacterium longum*, *Streptococcus thermophilus*	Placebo	8
Ayob et al. (28)	Malaysia	18/22	33.3	55.0	3 × 10^10^ CFU/d *Lactobacillus acidophilus* BCMC 12,130, *Lactobacillus casei* subsp. BCMC 12313, *Lactobacillus lactis* BCMC 12451, *Bifidobacterium bifidum* BCMC 02290, *Bifidobacterium infantis*, BCMC 02129, *Bifidobacterium longum* BCMC 02120	Placebo	24
Barcelos et al. (29)	Brazil	23/23	65.2	51.7	8 × 10^9^ CFU/d *Lactobacillus acidophilus* NCFM, *Lactobacillus rhamnosus* HN001, *Lactobacillus paracasei* LPC-37, *Bifidobacterium lactis* HN019	Placebo	24
Behrouz et al. (30)	Iran	30/30	26.7	38.5	5 × 10^9^ CFU/d *Lactobacillus casei*, *Lactobacillus rhamnosus*, *Lactobacillus acidophilus*, *Bifidobacterium longum*, *Bifidobacterium breve*	Placebo	12
Behrouz et al. (31)	Iran	30/30	26.7	38.5	5 × 10^9^ CFU/d *Lactobacillus casei*, *Lactobacillus rhamnosus*, *Lactobacillus acidophilus*, *Bifidobacterium longum*, *Bifidobacterium breve*	Placebo	12
Chong et al. (32)	UK	19/16	21.0	57.0	*streptococcus thermophilus*, *bifidobacterium breve*, *bifidobacterium longum*, *bifidobacterium infantis*, *lactobacillus acidophilus*, *lactobacillus plantarum*, *lactobacillus paracasei* and *lactobacillus bulgaricus*	Placebo	10
Derosa et al. (33)	Italy	30/30	50.0	55.8	*Streptococcus thermophilus* Bifidobacteria breve, Bifidobacteria animalis subspecies [subsp.] lactis BL03, Bifidobacteria animalis subsp. lactis BI04, *Lactobacillus acidophilus*, *Lactobacillus plantarum*, *Lactobacillus paracasei*, *Lactobacillus helveticus*	Placebo	12
Dinavari et al. (37)	Iran	40/40	55.0	49.4	2 × 10^9^ CFU/d *Bifidobacterium breve*, *Bifidobacterium longum*, Lacticaseibacillus rhamnosus, Lacticaseibacillus casei, *Lactobacillus bulgaricus*, *Lactobacillus acidophilus*, *Streptococcus thermophilus*	Placebo	12
Duseja et al. (34)	India	17/13	31.6	38.0	6.75 × 10^10^ CFU/d *Lactobacillus paracasei* DSM 24733, *Lactobacillus plantarum* DSM 24730, Lactobacillus acidophilus DSM 24735 and *Lactobacillus delbrueckii* subsp. bulgaricus DSM 24734, *Bifidobacterium longum* DSM 24736, *Bifidobacterium infantis* DSM 24737, *Bifidobacterium breve* DSM 24732, *Streptococcus thermophilus* DSM 24731	Placebo	48
Ekhlasi et al. (35)	Iran	15/15	/	/	2.8 × 10^9^ CFU/d *Lactobacillus casei*, *Lactobacillus rhamnosus*, Stretococcus thermophilus, *Bifidobacterium breve*, *Lactobacillus acidophilus*, *Bifidobacterium longum*, *Lactobacillus bulgaricus*	Placebo	8
Escouto et al. (36)	Brazil	23/25	87.0	58.0	2 × 10^9^ CFU/d *Lactobacillus acidophilus* ATCC SD5221, *Bifidobacterium lactis* HN019	Placebo	24
Ferolla et al. (38)	Brazil	27/23	76.0	57.3	10^8^ CFU/d *Lactobacillus reuteri*	Placebo	12
Hsieh et al. (39)	China	29/28	48.3	47.4	1.66 × 10^9^ CFU/d Bacillus coagulan TCI711	Placebo	8
Javadi et al. (41)	Iran	20/19	15.0	43.9	2 × 10^7^ CFU/d *Bifidobacterium longum*, *Lactobacillus acidophilus*	Placebo	12
Javadi 2017 et al. (41)	Iran	20/19	15.0	43.9	2 × 10^7^ CFU/d *Bifidobacterium longum*, *Lactobacillus acidophilus*	Placebo	12
Javadi et al. (42)	Iran	20/19	15.0	43.9	2 × 10^7^ CFU/d *Bifidobacterium longum*, *Lactobacillus acidophilus*	Placebo	12
Lin et al. (43)	China	15/11	/	/	2.01 × 10^10^ CFU/d *L. fermentum* TSF331, *L. reuteri* TSR332, *L. plantarum* TSP05	Placebo	8
Manzhalii et al. (44)	Ukraine	38/37	71.1	44.3	10^8^ CFU/d *Lactobacillus casei*, *Lactobacillus rhamnosus*, Lactobacillus bulgaris, *Bifidobacterium longum*, *Streptococcus thermophilus*	Placebo	12
Mitrović et al. (45)	Serbia	41/43	/	69	6.4 × 10^10^ CFU/d *Lactobacillus acidophilus* CBT LA1, *Lactobacillus casei* CBT LC5, *Bifidobacterium lactis* CBT BL3	Placebo	12
Mohamad et al. (46)	Malaysia	17/22	35.3	54.7	3 × 10^10^ CFU/d *Lactobacillus acidophilus* BCMC 12130, *Lactobacillus casei* subsp. BCMC 12313, *Lactobacillus lactis* BCMC 12451, Bifidobacterium bifidum BCMC 02290, *Bifidobacterium infantis* BCMC 02129, *Bifidobacterium longum* BCMC 02120	Placebo	24
Nabavi 2014 et al. (47)	Iran	36/36	52.8	42.8	2.481 × 10^9^ CFU/d *Lactobacillus acidophilus* La5, *Bifidobacterium lactis* Bb12	Placebo	8
Sadrkabir et al. (48)	Iran	33/28	33.3	43.3	1,000 mg Lactobacilli, cassia, acidophilous, bifidobacteria, streptococcus	Placebo	8
Sayari 2018 et al. (49)	Iran	70/68	44.1	43.4	7 × 10^9^ CFU/d *Lactobacillus casei*, *Lactobacillus rhamnosus*, *Lactobacillus acidophilus*, *Lactobacillus bulgaricus*, *Bifidobacterium breve*, *Bifidobacterium longum*, *Streptococcus thermophilus*	Placebo	16
Scorletti et al. (50)	UK	45/44	31.0	50.2	2 × 10^10^ CFU/d Bifidobacterium animalis subsp. lactis BB-12	Placebo	12
Sepideh et al. (51)	Iran	21/21	38.1	42.1	6.18 × 10^10^ CFU/d *Lactobacillus casei*, *Lactobacillus acidophilus*, *Lactobacillus rhamnosus*, *Lactobacillus bulgaricus*, *Bifidobacterium breve*, *Bifidobacterium longum*, *Streptococcus thermophilus*	Placebo	8
Shavakhi et al. (52)	Iran	31/32	54.8	41.5	1.9 × 10^9^ CFU/d *Lactobacillus acidophilus*, *Lactobacillus casei*, *Lactobacillus rhamnosus*, *Lactobacillus bulgaricus*, *Bifidobacterium breve*, *Bifidobacterium longum*, *Streptococcus thermophilus*	Placebo	24
Silva-Sperb et al. (26)	Brazil	23/23	65.0	51.0	8 × 10^9^ CFU/d *Lactobacillus acidophilus* NCFM, *Lactobacillus rhamnosus* HN001, *Lactobacillus paracasei* LPC-37, *Bifidobacterium lactis* HN019	Placebo	24
Won et al. (53)	Korea	80/23	/	/	9 × 10^9^ CFU/d *L. lactis* CKDB001	Placebo	8

E, experimental group; C, control group. There were no significant differences in female ratio, average age, or disease duration between the experimental and control groups in each included study.

Regarding specific trials, several included articles were identified as companion publications derived from the same clinical cohorts. These include multiple reports by Javadi et al. (three articles) (40–42), Behrouz et al. (two articles) (30, 31), the Malaysian cohort (Mohamad et al. and Ayob et al.) (28, 46) and the Brazilian PROBILIVER trial (Barcelos et al. and Silva-Sperb et al.) (26, 29). To prevent patient duplication in the statistical analysis, these companion publications were treated as single respective studies. Their data were consolidated to ensure that the same patient cohort contributed data only once to any single-outcome meta-analysis.

Regarding disease diagnosis, the vast majority of studies focused on patients with MASLD/NAFLD. However, 7 studies, namely Abhari et al. (24), Behrouz et al. (30), Behrouz et al. (31), Duseja et al. (34), Ekhlasi et al. (35), Javadi et al. (42), and Sadrkabir et al. (48), specifically excluded patients with concurrent diabetes. The mean age of the participants varied widely, primarily concentrating between 38 and 69 years. The proportion of female participants also differed across studies, ranging from 15 to 87%. Notably, within each individual study, there were no statistically significant differences between the experimental and control groups regarding the proportion of females, mean age, and disease duration, indicating excellent baseline comparability.

Across all included studies, the control groups uniformly utilized placebos as the comparator intervention. The experimental groups were administered a diverse range of probiotic preparations, encompassing both single-strain and multi-strain compound formulations. Additionally, several studies, including Abhari et al. (24), Asgharian et al. (27), Dinavari et al. (37), Ekhlasi et al. (35), Ferolla et al. (38), Javadi et al. (40), Javadi et al. (41), Javadi et al. (42), Manzhalii et al. (44), Mitrović et al. (45), Sadrkabir et al. (48), Sayari et al. (49), and Scorletti et al. (50), utilized synbiotic preparations. The core strains primarily involved the genera Lactobacillus, Bifidobacterium, and Streptococcus, as well as *Bacillus coagulans*. The daily intervention dosage of probiotics varied substantially, ranging from a minimum of 2 × 10^7^ CFU/d to a maximum of 6.75 × 10^10^ CFU/d. The established treatment durations ranged from a minimum of 8 weeks to a maximum of 48 weeks, with 8, 12, and 24 weeks being the most frequently adopted intervals.

### 3.3 Risk of bias

**Figure 2:**
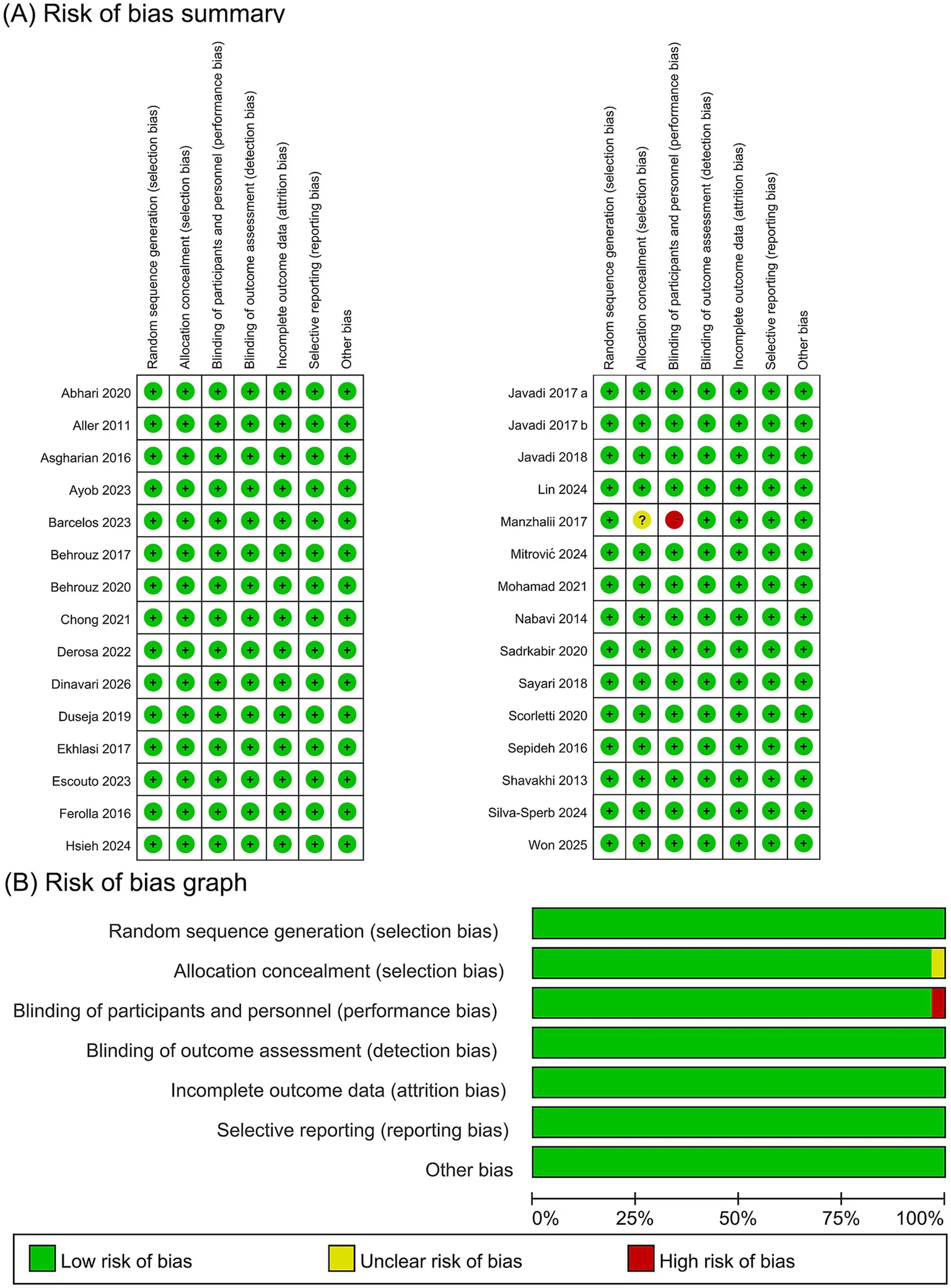
Risk of bias assessment of the included studies. Quality assessment was performed using the Cochrane Risk of Bias tool. **(A)** Risk of bias summary: Judgments about each risk of bias item for each included study (green indicates low risk, yellow indicates unclear risk, and red indicates high risk). **(B)** Risk of bias graph: Review authors’ judgments about each risk of bias item presented as percentages across all included studies.

In this meta-analysis, the vast majority of the included studies utilized computer-generated random sequences and implemented allocation concealment, demonstrating a low risk of selection bias. All studies employed a rigorous placebo-controlled design, which effectively mitigated potential performance bias related to the blinding of participants and personnel. Notably, the study by Manzhalii et al. (44) lacked clear reporting on the specific procedures for allocation concealment and failed to effectively blind participants and study personnel. Regarding outcome assessment, the risk of detection bias was considered low across all studies because all outcome indicators relied on objective laboratory data. Furthermore, the attrition rate in all studies was below 20%, indicating a low risk of attrition bias arising from incomplete outcome data. Every study comprehensively reported its pre-defined outcomes, and no evidence of selective reporting or other apparent biases was observed. Overall, the included literature exhibited good methodological quality, with the risk of bias remaining low across the majority of domains. A detailed summary of the risk of bias is presented in Figure 2.

### 3.4 Meta-analysis results

#### 3.4.1 Effects of probiotics and synbiotics on liver enzymes and liver injury

**Figure 3:**
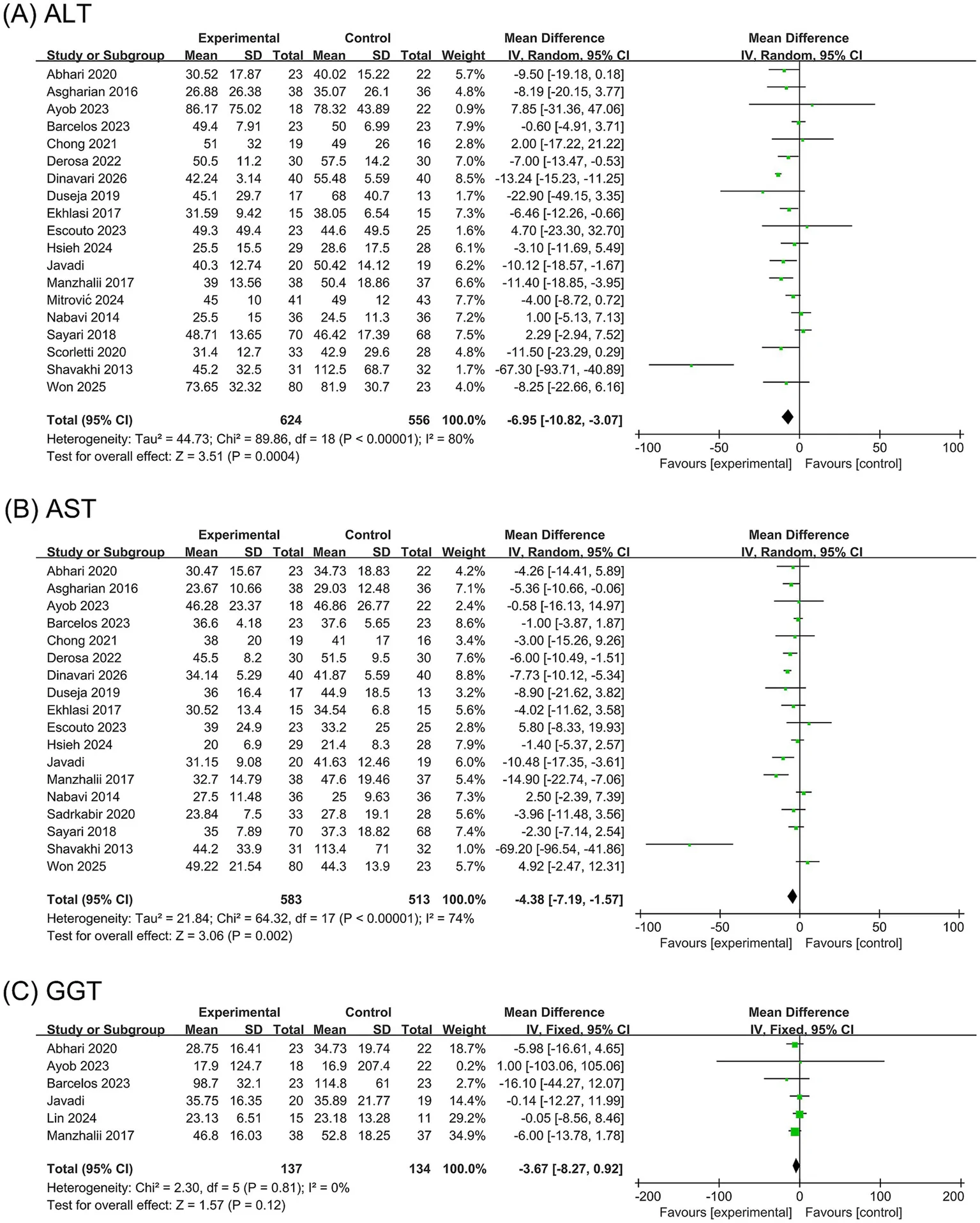
Forest plots of the effects of probiotics on liver enzymes in patients with MASLD. Forest plots detailing the mean difference and 95% confidence intervals (CIs) for **(A)** alanine aminotransferase (ALT), **(B)** aspartate aminotransferase (AST), and **(C)** gamma-glutamyl transferase (GGT). The size of the squares represents the weight of each study, and the black diamond represents the pooled overall effect.

ALT: The meta-analysis included 19 studies (24, 27–29, 32–37, 39–41, 44, 45, 47, 49, 50, 52, 53), involving a total of 1,180 participants. Cochran’s *Q* test and the *I*^2^ statistic revealed high heterogeneity (*p* < 0.00001, *I*^2^ = 80%); therefore, a random-effects model was employed for data pooling. The results demonstrated that compared with the placebo group, the probiotic group exhibited significantly reduced ALT levels (MD = −6.95 U/L, 95% CI: −10.82 to −3.07, *p* = 0.0004), as shown in Figure 3A.

AST: The meta-analysis included 18 studies (24, 27–29, 32–37, 39–41, 44, 47–49, 52, 53), comprising 1,096 participants. Cochran’s *Q* test and the *I*^2^ statistic indicated high heterogeneity (*p* < 0.00001, *I*^2^ = 74%); thus, a random-effects model was utilized to synthesize the data. The findings showed a significant reduction in AST levels in the probiotic group compared to the placebo group (MD = −4.38 U/L, 95% CI: −7.19 to −1.57, *p* = 0.002), as depicted in Figure 3B.

GGT: The meta-analysis included 6 studies (24, 28, 29, 40, 41, 43, 44) with 271 participants. Cochran’s *Q* test and the *I*^2^ statistic showed very low heterogeneity (*p* = 0.81, *I*^2^ = 0); consequently, a fixed-effects model was applied for data pooling. The results indicated that there was no statistically significant effect of probiotics on reducing GGT levels compared with the placebo group (MD = −3.67 U/L, 95% CI: −8.27 to 0.92, *p* = 0.12), as illustrated in Figure 3C.

#### 3.4.2 Effects of probiotics and synbiotics on lipid metabolism profiles

**Figure 4:**
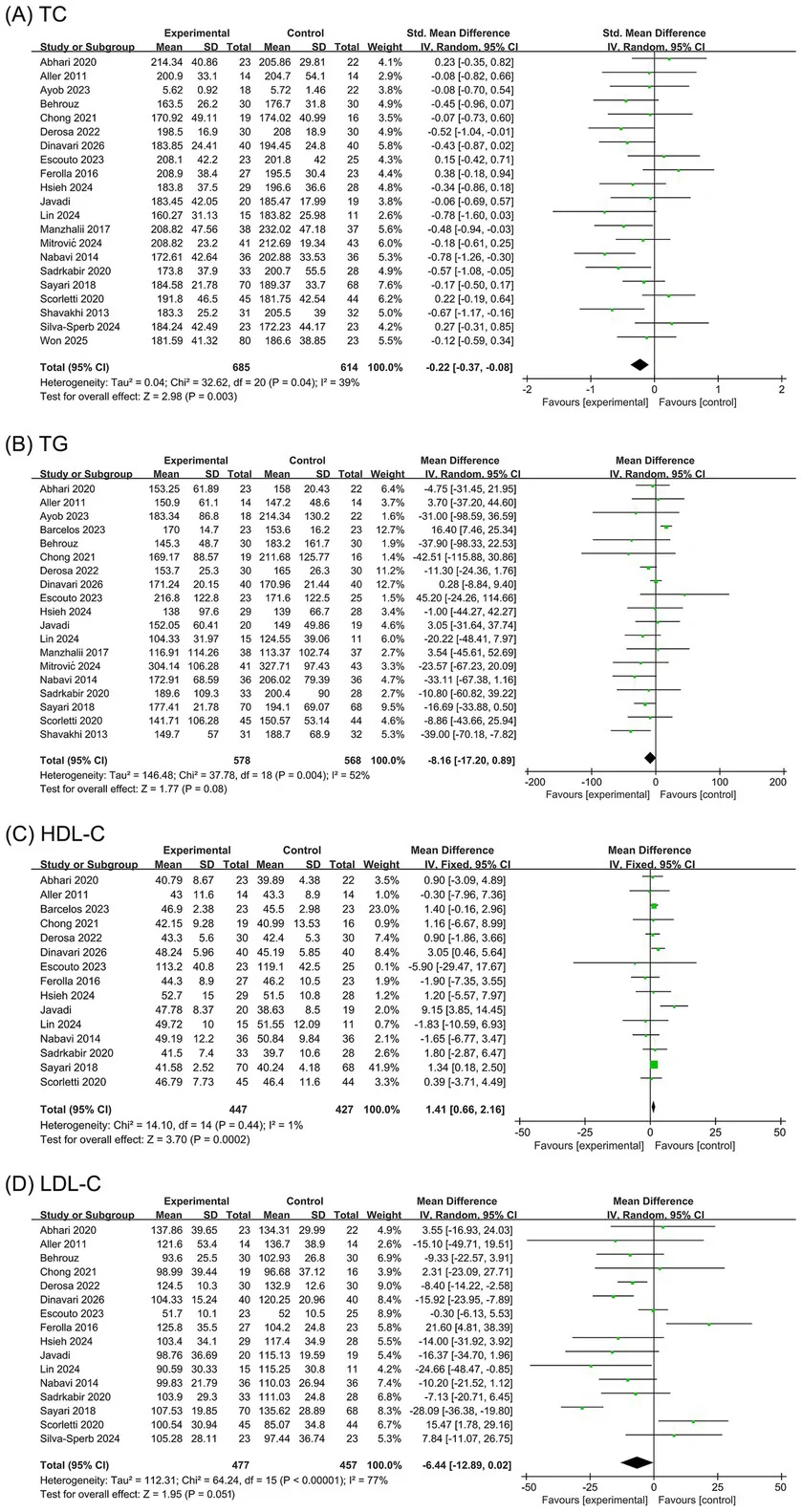
Forest plots of the effects of probiotics on lipid profiles in patients with MASLD. Forest plots detailing the mean difference and 95% CIs for **(A)** total cholesterol (TC), **(B)** triglycerides (TG), **(C)** high-density lipoprotein cholesterol (HDL-C), and **(D)** low-density lipoprotein cholesterol (LDL-C).

TC: The meta-analysis included 21 studies (24–26, 28, 31–33, 36–41, 43–45, 47–50, 52, 53), involving a total of 1,299 participants. Cochran’s *Q* test and the *I*^2^ statistic revealed high heterogeneity (*p* = 0.04, *I*^2^ = 39%), prompting the use of a random-effects model for data pooling. The results indicated that there was a statistically significant effect of probiotics on reducing TC levels compared with the placebo group (SMD = −0.22, 95% CI: −0.37 to −0.08, *p* = 0.003), as shown in Figure 4A.

TG: The meta-analysis included 19 studies (24, 25, 28, 29, 31–33, 36, 37, 39–41, 43–45, 47–50, 52), involving a total of 1,146 participants. Cochran’s *Q* test and the *I*^2^ statistic showed moderate heterogeneity (*p* = 0.004, *I*^2^ = 52%); therefore, a random-effects model was employed for data pooling. The results indicated that there was no statistically significant effect of probiotics on reducing TG levels compared with the placebo group (MD = −8.16 mg/dL, 95% CI: −17.20 to 0.89, *p* = 0.08), as shown in Figure 4B.

HDL-C: The meta-analysis included 15 studies (24, 25, 29, 32, 33, 36–41, 43, 47–50), comprising 874 participants. Cochran’s *Q* test and the *I*^2^ statistic indicated very low heterogeneity (*p* = 0.44, *I*^2^ = 1%); thus, a fixed-effects model was utilized to synthesize the data. The findings demonstrated that the probiotic group significantly increased HDL-C levels compared to the placebo group (MD = 1.41 mg/dL, 95% CI: 0.66 to 2.16, *p* = 0.0002), as depicted in Figure 4C.

LDL-C: The meta-analysis included 16 studies (24–26, 31–33, 36–41, 43, 47–50), involving a total of 934 participants. Cochran’s *Q* test and the *I*^2^ statistic revealed high heterogeneity (*p* < 0.00001, *I*^2^ = 77%), prompting the use of a random-effects model for data pooling. The results showed that there was no statistically significant effect of probiotics on reducing LDL-C levels compared with the placebo group (MD = −6.44 mg/dL, 95% CI: −12.89 to 0.02, *p* = 0.051), as illustrated in Figure 4D.

#### 3.4.3 Effects of probiotics and synbiotics on glycemic control and insulin resistance

**Figure 5:**
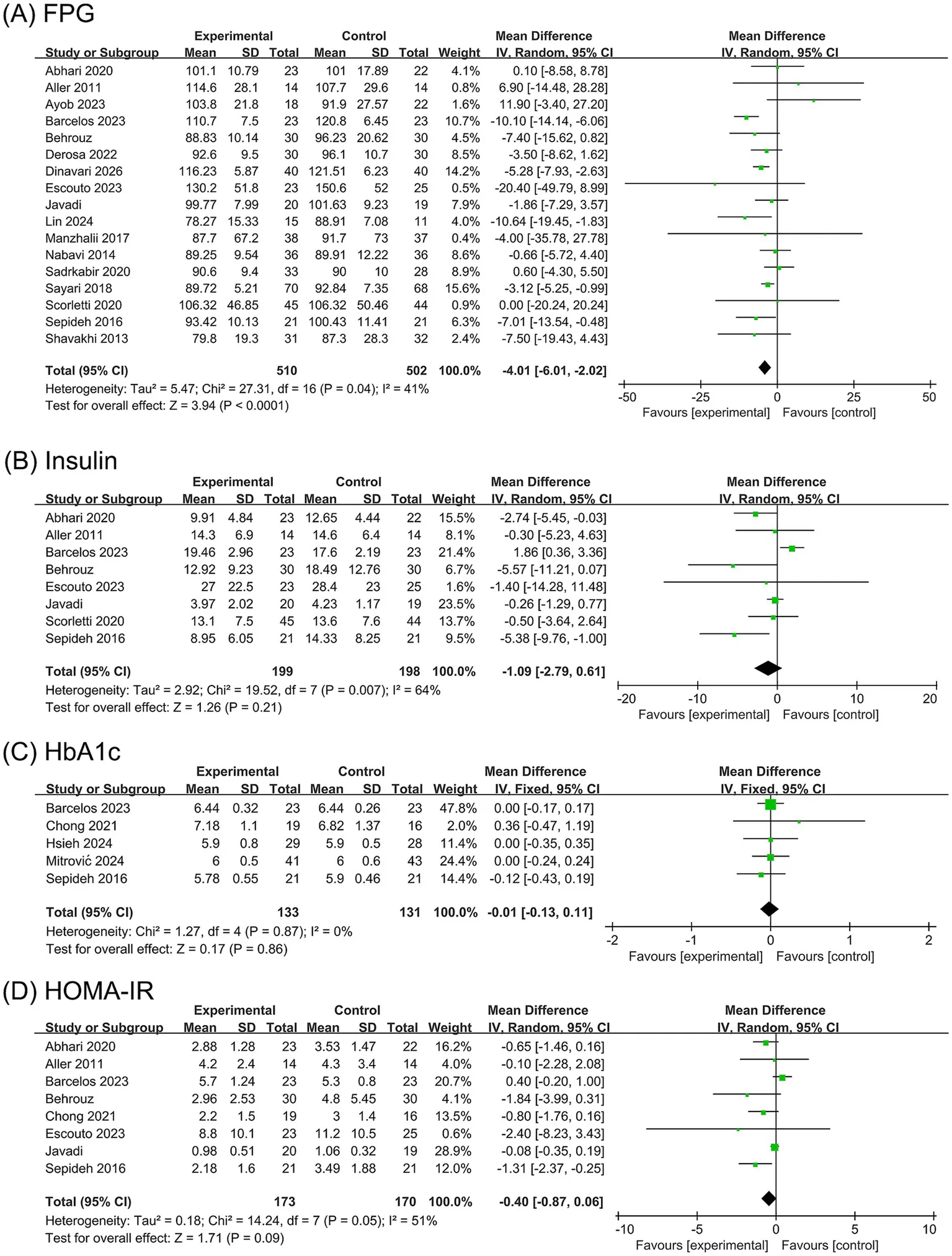
Forest plots of the effects of probiotics on glycemic control and insulin resistance. Forest plots detailing the mean difference and 95% CIs for **(A)** fasting plasma glucose (FPG), **(B)** insulin, **(C)** glycated hemoglobin (HbA1c), and **(D)** homeostatic model assessment for insulin resistance (HOMA-IR).

FPG: The meta-analysis included 17 studies (24, 25, 28–30, 33, 36, 37, 40, 41, 43, 44, 47–52), involving a total of 1,012 participants. Cochran’s *Q* test and the *I*^2^ statistic showed moderate heterogeneity (*p* = 0.04, *I*^2^ = 41%); therefore, a random-effects model was employed for data pooling. The results demonstrated that compared with the placebo group, the probiotic group exhibited significantly reduced FPG levels (MD -4.01 mg/dL, 95% CI: −6.01 to −2.02, *p* < 0.0001), as shown in Figure 5A.

Insulin: The meta-analysis included 8 studies (24, 25, 29, 30, 36, 40, 41, 50, 51), comprising 397 participants. Cochran’s *Q* test and the *I*^2^ statistic indicated high heterogeneity (*p* = 0.007, *I*^2^ = 64%); thus, a random-effects model was utilized to synthesize the data. The results indicated that there was no statistically significant effect of probiotics on reducing insulin levels compared with the placebo group (MD = −1.09 U/L, 95% CI: −2.79 to 0.61, *p* = 0.21), as depicted in Figure 5B.

HbA1c: The meta-analysis included 5 studies (29, 32, 39, 45, 51) with 264 participants. Cochran’s *Q* test and the *I*^2^ statistic showed very low heterogeneity (*p* = 0.87, *I*^2^ = 0); consequently, a fixed-effects model was applied for data pooling. The findings demonstrated no statistically significant effect of probiotics on reducing HbA1c levels compared with the placebo group (MD = −0.01, 95% CI: −0.13 to 0.11, *p* = 0.86), as illustrated in Figure 5C.

HOMA-IR: The meta-analysis included 8 studies (24, 25, 29, 30, 32, 36, 40, 41, 51), involving a total of 343 participants. Cochran’s *Q* test and the *I*^2^ statistic revealed moderate heterogeneity (*p* = 0.05, *I*^2^ = 51%), prompting the use of a random-effects model for data pooling. The results indicated that the probiotic group showed a decreasing trend in HOMA-IR compared to the placebo group, although this did not reach statistical significance (MD = −0.40, 95% CI: −0.87 to 0.06, *p* = 0.09), as shown in Figure 5D.

#### 3.4.4 Effects of probiotics and synbiotics on systemic inflammation

**Figure 6:**
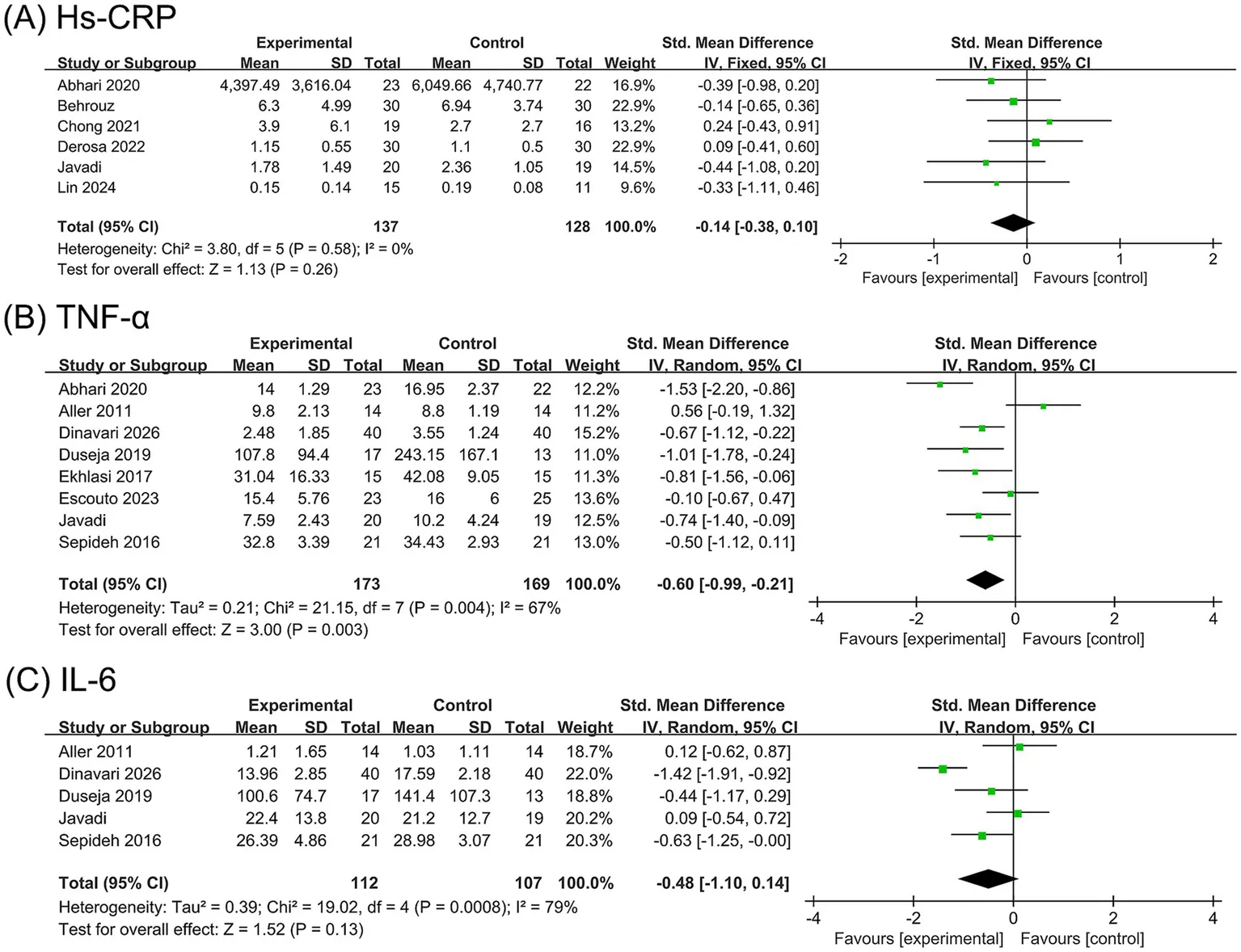
Forest plots of the effects of probiotics on systemic inflammatory markers. Forest plots detailing the effect sizes and 95% CIs for **(A)** high-sensitivity C-reactive protein (Hs-CRP), **(B)** tumor necrosis factor-alpha (TNF-α), and **(C)** interleukin-6 (IL-6).

hs-CRP: The meta-analysis included 6 studies (24, 31–33, 42, 43), comprising 265 participants. Cochran’s *Q* test and the *I*^2^ statistic indicated very low heterogeneity (*p* = 0.58, *I*^2^ = 0); thus, a fixed-effects model was utilized to synthesize the data. The results showed that there was no statistically significant effect of probiotics on reducing hs-CRP levels compared with the placebo group (SMD = −0.14, 95% CI: −0.38 to 0.10, *p* = 0.26), as depicted in Figure 6A.

TNF-α: The meta-analysis included 8 studies (24, 25, 27, 34–36, 42, 51), involving a total of 342 participants. Cochran’s *Q* test and the *I*^2^ statistic revealed high heterogeneity (*p* = 0.004, *I*^2^ = 67%); therefore, a random-effects model was employed for data pooling. The findings demonstrated that the probiotic group significantly reduced TNF-α levels compared to the placebo group (SMD = −0.60, 95% CI: −0.99 to −0.21, *p* = 0.003), as illustrated in Figure 6B.

IL-6: The meta-analysis included 5 studies (25, 34, 37, 42, 51) with 219 participants. Cochran’s *Q* test and the *I*^2^ statistic revealed high heterogeneity (*p* = 0.0008, *I*^2^ = 79%); therefore, a random-effects model was employed for data pooling. The results showed that there was no statistically significant effect of probiotics on reducing IL-6 levels compared with the placebo group (SMD = −0.48, 95% CI: −1.10 to 0.14, *p* = 0.13), as shown in Figure 6C.

### 3.5 Sensitivity analysis

The aforementioned analyses indicated moderate heterogeneity for TC and FPG (25% < *I*^2^ ≤ 50%), whereas ALT, AST, TG, LDL-C, insulin, HOMA-IR, TNF-α, and IL-6 exhibited high heterogeneity (*I*^2^ > 50%). Consequently, a leave-one-out sensitivity analysis was conducted to explore the sources of heterogeneity for these outcomes, as detailed in Table 2.

**Table 2:** Leave-one-out sensitivity analyses of identified heterogeneous sources.

Outcome	Heterogeneity source	Sensitivity analysis results			Robustness
		*I*^2^/%	MD/SMD (95% CI)	*p-*value	
TG	Barcelos et al. (30)	0	−8.27 (−13.99, −2.55)	0.005	Not robust
HOMA-IR	Sepideh et al. (51)	37%	−0.23 (−0.65, 0.18)	0.27	Robust
IL-6	Dinavari et al. (37)	20%	−0.22 (−0.60, 0.15)	0.25	Robust

MD, mean difference; CI, confidence interval; TC, total cholesterol; HOMA-IR, homeostasis model assessment of insulin resistance; IL-6, interleukin 6.

The sensitivity analysis revealed that the heterogeneity observed for TG was associated with the study by Barcelos et al. (29), potentially because this trial imposed no restrictions on participant body weight. Upon the exclusion of this study, the heterogeneity for TG was substantially eliminated (*I*^2^ = 0), and the pooled effect became statistically significant (MD = −8.27 mg/dL, 95% CI: −13.99 to −2.55, *p* = 0.005), indicating that the initial meta-analysis results for TG were not robust. The heterogeneity for HOMA-IR was linked to the study by Sepideh et al. (51). This could be attributed to the study’s broader inclusion criteria for participants, the specific probiotic dosage utilized, or an insufficient intervention duration; notably, the probiotic dosage administered in this trial was considerably higher than that in the other included studies. Following the exclusion of this study, the heterogeneity for HOMA-IR was markedly reduced (*I*^2^ = 37%), while the statistical significance remained unchanged (MD = −0.23, 95% CI: −0.65 to 0.18, *p* = 0.27), suggesting that the meta-analysis results for HOMA-IR are robust. The heterogeneity for IL-6 was linked to the study by Dinavari et al. (37). This may be attributable to the use of synbiotics containing both probiotics and prebiotics, whereas other studies employed only pure probiotics as the intervention. Following the exclusion of this study, the heterogeneity for IL-6 was markedly reduced (*I*^2^ = 20%), while the statistical significance remained unchanged (SMD = −0.22, 95% CI: −0.60 to 0.15, *p* = 0.25), suggesting that the meta-analysis results for IL-6 are robust.

Furthermore, the sensitivity analysis demonstrated that after the sequential omission of individual studies, the direction of the pooled effect sizes for AST, ALT, TC, insulin, FPG, LDL-C, and TNF-α did not change. Additionally, the heterogeneity statistic (*I*^2^) did not undergo any substantial decrease, indicating that no single study was the decisive driver of the overall effect estimates. This analytical pattern suggests that the heterogeneity observed in the pooled results for TC and LDL-C does not stem from individual outliers. Instead, it is more likely attributable to pervasive, multifactorial differences across the included studies. These factors include, but are not limited to: inherent variations in the baseline TC and LDL-C levels of the study populations, disparities in the concurrent use of lipid-lowering medications, inconsistencies in the composition and dosage of probiotic strains, and a lack of standardization in dietary control and monitoring protocols during the intervention periods across the various trials.

### 3.6 Subgroup analysis

Subgroup analyses were conducted to investigate how clinical factors (e.g., participant region, mean age, diabetes comorbidity, treatment duration, and intervention program) influenced the high heterogeneity observed in the levels of ALT, AST, TC, TG, LDL-C, FPG, insulin, HOMA-IR, TNF-α, and IL-6 as detailed in Table 3.

**Table 3:** Subgroup analyses of the high heterogeneity outcomes.

Outcome	Subject	Subgroup	Number of studies	*I*^2^/%	SMD/MD (95% CI)	*p-*value
ALT	Participant source	Asia	13	82	−8.57 (−13.87, −3.27)	0.002
		Europe	4	13	−6.25 (−9.99, −2.50)	0.001
		South America	2	0	−0.48 (−4.74, 3.79)	0.83
	Average age	<50 years old	11	85	−9.57 (−15.33, −3.81)	0.001
		≥50 years old	6	0	−2.85 (−5.66, −0.05)	0.047
	Comorbidities	Diabetes-excluded	4	0	−8.35 (−12.87, −3.83)	0.0003
		Diabetes-included	15	84	−6.29 (−10.92, −1.66)	0.008
	Treatment duration	<24 weeks	14	76	−6.36 (−10.02, −2.70)	0.0007
		≥24 weeks	5	85	−15.93 (−41.08, 9.23)	0.21
	Intervention program	Synbiotics	9	80	−7.73 (−12.16, −3.30)	0.0006
		Probiotics	10	71	−6.15 (−12.77, 0.48)	0.07
AST	Participant source	Asia	14	76	−4.39 (−7.94, −0.84)	0.02
		Europe	3	54	−8.33 (−14.85, −1.81)	0.01
		South America	2	0	−0.73 (−3.54, 2.08)	0.61
	Average age	<50 years old	11	79	−6.44 (−10.36, −2.51)	0.001
		≥50 years old	5	15	−2.38 (−5.34, 0.57)	0.11
	Comorbidities	Diabetes-excluded	4	0	−4.64 (−9.07, −0.20)	0.04
		Diabetes-included	14	80	−4.33 (−7.66, −1.01)	0.01
	Treatment duration	<24 weeks	13	65	−4.26 (−6.90, −1.62)	0.002
		≥24 weeks	5	85	−9.94 (−23.62, 3.74)	0.15
	Intervention program	Synbiotics	8	35	−6.55 (−9.06, −4.04)	<0.0001
		Probiotics	10	75	−2.37 (−6.71, 1.97)	0.28
TC	Participant source	Asia	12	21	−0.34 (−0.51, −0.18)	<0.0001
		Europe	6	30	−0.19 (−0.44, 0.07)	0.15
		South America	3	0	0.27 (−0.06, 0.60)	0.11
	Average age	<50 years old	11	17	−0.37 (−0.53, −0.20)	<0.0001
		≥50 years old	8	19	0.02 (−0.19, 0.22)	0.88
	Comorbidities	Diabetes-excluded	3	56	−0.28 (−0.75, 0.19)	0.24
		Diabetes-included	18	39	−0.21 (−0.37, −0.05)	0.009
	Treatment duration	<24 weeks	17	36	−0.24 (−0.40, −0.09)	0.002
		≥24 weeks	4	57	−0.10 (−0.53, 0.34)	0.66
	Intervention program	Synbiotics	9	44	−0.14 (−0.35, 0.07)	0.21
		Probiotics	12	32	−0.30 (−0.50, −0.10)	0.003
TG	Participant source	Asia	11	21	−11.70 (−20.94, −2.47)	0.01
		Europe	6	0	−10.72 (−21.62, 0.18)	0.054
		South America	2	0	16.87 (8.00, 25.74)	0.0002
	Average age	<50 years old	11	15	−8.83 (−17.64, −0.02)	0.049
		≥50 years old	7	67	−3.91 (−22.71, 14.89)	0.68
	Comorbidities	Diabetes-excluded	3	0	−10.29 (−32.23, 11.66)	0.36
		Diabetes-included	16	58	−7.91 (−17.88, 2.05)	0.12
	Treatment duration	<24 weeks	15	0	−7.36 (−13.20, −1.53)	0.01
		≥24 weeks	4	78	−3.43 (−41.80, 34.94)	0.86
	Intervention program	Synbiotics	8	0	−4.03 (−11.13, 3.08)	0.27
		Probiotics	11	69	−11.79 (−28.10, 4.52)	0.16
LDL-C	Participant source	Asia	9	50	−14.48 (−20.76, −8.21)	<0.00001
		Europe	4	72	−0.03 (−15.26, 15.20)	1.00
		South America	3	68	8.05 (−5.69, 21.79)	0.25
	Average age	<50 years old	9	48	−13.97 (−20.25, −7.69)	<0.0001
		≥50 years old	6	75	4.68 (−4.42, 13.78)	0.31
	Comorbidities	Diabetes-excluded	3	0	−6.18 (−14.78, 2.43)	0.16
		Diabetes-included	13	81	−6.67 (−14.29, 0.95)	0.09
	Treatment duration	<24 weeks	14	75	−7.92 (−15.07, −0.77)	0.03
		≥24 weeks	2	0	0.41 (−5.16, 5.98)	0.89
	Intervention program	Synbiotics	7	88	−4.61 (−18.33, 9.11)	0.51
		Probiotics	9	27	−6.27 (−11.07, −1.47)	0.01
FPG	Participant source	Asia	11	36	−3.33 (−5.35, −1.30)	0.001
		Europe	4	0	−2.80 (−7.58, 1.98)	0.25
		South America	2	0	−10.29 (−14.30, −6.29)	<0.00001
	Average age	<50 years old	11	0	−3.37 (−4.74, −1.99)	<0.00001
		≥50 years old	5	64	−4.26 (−11.40, 2.87)	0.24
	Comorbidities	Diabetes-excluded	3	28	−1.57 (−6.30, 3.17)	0.54
		Diabetes-included	14	41	−4.54 (−6.70, −2.37)	<0.0001
	Treatment duration	<24 weeks	13	2	−3.47 (−4.82, −2.11)	<0.00001
		≥24 weeks	4	63	−5.51 (−15.64, 4.61)	0.29
	Intervention program	Synbiotics	7	0	−3.25 (−4.73, −1.77)	<0.00001
		Probiotics	10	50	−5.39 (−9.00, −1.78)	0.003
Insulin	Participant source	Asia	4	70	−2.73 (−5.43, −0.03)	0.047
		Europe	2	0	−0.44 (−3.09, 2.20)	0.74
		South America	2	0	1.82 (0.32, 3.31)	0.02
	Average age	<50 years old	5	60	−2.27 (−4.53, −0.02)	0.049
		≥50 years old	3	0	1.39 (0.04, 2.74)	0.04
	Comorbidities	Diabetes-excluded	2	0	−3.27 (−5.72, −0.83)	0.009
		Diabetes-included	6	58	−0.26 (−1.97, 1.45)	0.76
	Treatment duration	<24 weeks	6	50	−1.81 (−3.59, −0.03)	0.047
		≥24 weeks	2	0	1.82 (0.32, 3.31)	0.02
	Intervention program	Synbiotics	3	29	−0.81 (−2.20, 0.58)	0.25
		Probiotics	5	73	−1.88 (−5.78, 2.02)	0.35
HOMA-IR	Participant source	Asia	4	64	−0.67 (−1.38, 0.04)	0.07
		Europe	2	0	−0.69 (−1.57, 0.19)	0.13
		South America	2	0	0.37 (−0.23, 0.97)	0.23
	Average age	<50 years old	5	53	−0.59 (−1.22, 0.03)	0.06
		≥50 years old	3	60	−0.21 (−1.30, 0.88)	0.71
	Comorbidities	Diabetes-excluded	2	3	−0.81 (−1.61, −0.01)	0.047
		Diabetes-included	6	52	−0.28 (−0.79, 0.24)	0.29
	Treatment duration	<24 weeks	6	49	−0.25 (−0.49, −0.02)	0.03
		≥24 weeks	2	0	0.37 (−0.23, 0.97)	0.23
	Intervention program	Synbiotics	2	42	−0.23 (−0.73, 0.26)	0.36
		Probiotics	6	59	−0.63 (−1.50, 0.23)	0.15
TNF-α	Participant source	Asia	6	16	−0.84 (−1.12, −0.56)	<0.00001
		Europe	1	0	0.56 (−0.19, 1.32)	0.15
		South America	1	0	−0.10 (−0.67, 0.47)	0.73
	Average age	<50 years old	6	71	−0.66 (−1.15, −0.17)	0.008
		≥50 years old	1	0	−0.10 (−0.67, 0.47)	0.73
	Comorbidities	Diabetes-excluded	4	6	−1.03 (−1.39, −0.67)	<0.00001
		Diabetes-included	4	65	−0.23 (−0.73, 0.26)	0.35
	Treatment duration	<24 weeks	6	70	−0.63 (−1.11, −0.15)	0.009
		≥24 weeks	2	71	−0.51 (−1.40, 0.37)	0.26
	Intervention program	Synbiotics	4	35	−0.91 (−1.29, −0.53)	<0.00001
		Probiotics	4	67	−0.26 (−0.84, 0.32)	0.38
IL-6	Participant source	Asia	4	80	−0.62 (−1.30, 0.06)	0.07
		Europe	1	0	0.12 (−0.62, 0.87)	0.74
	Comorbidities	Diabetes-excluded	2	13	−0.14 (−0.66, 0.37)	0.59
		Diabetes-included	3	84	−0.67 (−1.55, 0.20)	0.13
	Treatment duration	<24 weeks	4	84	−0.48 (−1.25, 0.28)	0.22
		≥24 weeks	1	0	−0.44 (−1.17, 0.29)	0.24
	Intervention program	Synbiotics	2	93	−0.68 (−2.15, 0.80)	0.37
		Probiotics	3	16	−0.35 (−0.78, 0.09)	0.12

MD, mean difference; CI, confidence interval; LDL-C, low-density lipoprotein cholesterol.

Regarding regional differences, both the Asian group (MD = −8.57 U/L, 95% CI: −13.87 to −3.27, *p* = 0.002, *I*^2^ = 82%) and the European group (MD = −6.25 U/L, 95% CI: −9.99 to −2.50, *p* = 0.001, *I*^2^ = 13%) showed a significant reduction in ALT levels. In contrast, no significant effect was observed in the South American group (MD = −0.48 U/L, 95% CI: −4.74 to 3.79, *p* = 0.83, *I*^2^ = 0). In terms of mean age, the probiotic intervention group aged <50 years exhibited a significant decrease in ALT levels (MD = −9.57 U/L, 95% CI: −15.33 to −3.81, *p* = 0.001, *I*^2^ = 85%), and the group aged ≥50 years also demonstrated a significant reduction (MD = −2.85 U/L, 95% CI: −5.66 to −0.05, *p* = 0.047, *I*^2^ = 0). Concerning diabetes comorbidity, both the diabetes-excluded group (MD = −8.35 U/L, 95% CI: −12.87 to −3.83, *p* = 0.0003, *I*^2^ = 0) and the non-excluded group (MD = −6.29 U/L, 95% CI: −10.92 to −1.66, *p* = 0.008, *I*^2^ = 84%) demonstrated significantly reduced ALT levels. Based on treatment duration, significant reductions were observed in the <24 weeks intervention group (MD = −6.36 U/L, 95% CI: −10.02 to −2.70, *p* = 0.0007, *I*^2^ = 76%), whereas the ≥24 weeks group showed no significant effect (MD = −15.93 U/L, 95% CI: −41.08 to 9.23, *p* = 0.21, *I*^2^ = 85%). Regarding the intervention program, the synbiotics group showed a significant reduction in ALT levels (MD = −7.73 U/L, 95% CI: −12.16 to −3.30, *p* = 0.0006, *I*^2^ = 80%), whereas the probiotics group demonstrated no significant effect (MD = −6.15 U/L, 95% CI: −12.77 to 0.48, *p* = 0.07, *I*^2^ = 71%). In summary, the heterogeneity of ALT appears to be associated with participant region, mean age, and whether diabetes was excluded, but it is not related to treatment duration or intervention program.

AST: In terms of regional differences, both Asian participants (MD = −4.39 U/L, 95% CI: −7.94 to −0.84, *p* = 0.02, *I*^2^ = 76%) and European participants (MD = −8.33 U/L, 95% CI: −14.85 to −1.81, *p* = 0.01, *I*^2^ = 54%) showed a significant decrease in AST levels, while no significant effect was observed in South American participants (MD = −0.73 U/L, 95% CI: −3.54 to 2.08, *p* = 0.61, *I*^2^ = 0). Regarding mean age, the <50 years probiotic group significantly reduced AST levels (MD = −6.44 U/L, 95% CI: −10.36 to −2.51, *p* = 0.001, *I*^2^ = 79%), whereas the ≥50 years group demonstrated no significant effect (MD = −2.38 U/L, 95% CI: −5.34 to 0.57, *p* = 0.11, *I*^2^ = 15%). Concerning diabetes comorbidity, both the diabetes-excluded group (MD = −4.64 U/L, 95% CI: −9.07 to −0.20, *p* = 0.04, *I*^2^ = 0) and the non-excluded group (MD = −4.33 U/L, 95% CI: −7.66 to −1.01, *p* = 0.01, *I*^2^ = 80%) significantly lowered AST levels. Regarding treatment duration, interventions lasting <24 weeks significantly reduced AST levels (MD = −4.26 U/L, 95% CI: −6.90 to −1.62, *p* = 0.002, *I*^2^ = 65%), while interventions lasting ≥24 weeks showed no significant effect (MD = −9.94 U/L, 95% CI: −23.62 to 3.74, *p* = 0.15, *I*^2^ = 85%). Based on the intervention program, the synbiotics group exhibited a significant decrease in AST levels (MD = −6.55 U/L, 95% CI: −9.06 to −4.04, *p* < 0.0001, *I*^2^ = 35%), while the probiotics group demonstrated no significant effect (MD = −2.37 U/L, 95% CI: −6.71 to 1.97, *p* = 0.28, *I*^2^ = 75%). In conclusion, the heterogeneity of AST appears to be related to participant region, mean age, whether diabetes was excluded, and intervention program, but it is not associated with treatment duration.

TC: Subgroup analysis by study region revealed that Asian participants (SMD = −0.34, 95% CI: −0.51 to −0.18, *p* < 0.0001, *I*^2^ = 21%) showed a significant reduction in TC levels. However, European participants (SMD = −0.19, 95% CI: −0.44 to 0.07, *p* = 0.15, *I*^2^ = 30%) and South American participants (SMD = 0.27, 95% CI: −0.06 to 0.60, *p* = 0.11, *I*^2^ = 0) showed no statistically significant effect. Regarding mean age, the probiotic group aged <50 years demonstrated a significant decrease in TC levels (SMD = −0.37, 95% CI: −0.53 to −0.20, *p* < 0.0001, *I*^2^ = 17%), whereas the group aged ≥50 years showed no significant effect (SMD = 0.02, 95% CI: −0.19 to 0.22, *p* = 0.88, *I*^2^ = 19%). Concerning diabetes, the non-excluded group exhibited a significant decrease in TC levels (SMD = −0.21, 95% CI: −0.37 to −0.05, *p* = 0.009, *I*^2^ = 39%), whereas the diabetes-excluded group showed no significant effect (SMD = −0.28, 95% CI: −0.75 to 0.19, *p* = 0.24, *I*^2^ = 56%). Based on treatment duration, the <24 weeks intervention group significantly reduced TC levels (SMD = −0.24, 95% CI: −0.40 to −0.09, *p* = 0.002, *I*^2^ = 36%), while the ≥24 weeks group showed no significant effect (SMD = −0.10, 95% CI: −0.53 to 0.34, *p* = 0.66, *I*^2^ = 57%). Regarding the intervention program, the probiotics group significantly reduced TC levels (SMD = −0.30, 95% CI: −0.50 to −0.10, *p* = 0.003, *I*^2^ = 32%), whereas the synbiotics group showed no significant effect (SMD = −0.14, 95% CI: −0.35 to 0.07, *p* = 0.21, *I*^2^ = 44%). In summary, the heterogeneity of TC appears to be associated with participant region, mean age, intervention duration, whether diabetes was excluded, and intervention program.

TG: In terms of regional differences, the Asian group significantly reduced TG levels (MD = −11.70 mg/dL, 95% CI: −20.94 to −2.47, *p* = 0.01, *I*^2^ = 21%), while the South American group significantly increased TG levels (MD = 16.87 mg/dL, 95% CI: 8.00 to 25.74, *p* = 0.0002, *I*^2^ = 0). The European group showed no significant effect (MD = −10.72 mg/dL, 95% CI: −21.62 to 0.18, *p* = 0.054, *I*^2^ = 0). Regarding mean age, the <50 years group showed a significant reduction (MD = −8.83 mg/dL, 95% CI: −17.64 to −0.02, *p* = 0.049, *I*^2^ = 15%), whereas the ≥50 years group did not (MD = −3.91 mg/dL, 95% CI: −22.71 to 14.89, *p* = 0.68, *I*^2^ = 67%). Concerning the exclusion of diabetes, neither the diabetes-excluded group (MD = −10.29 mg/dL, 95% CI: −32.23 to 11.66, *p* = 0.36, *I*^2^ = 0) nor the non-excluded group (MD = −7.91 mg/dL, 95% CI: −17.88 to 2.05, *p* = 0.12, *I*^2^ = 58%) showed a significant effect. Based on treatment duration, the <24 weeks intervention group significantly reduced TG levels (MD = −7.36 mg/dL, 95% CI: −13.20 to −1.53, *p* = 0.01, *I*^2^ = 0), whereas the ≥24 weeks group showed no significant effect (MD = −3.43 mg/dL, 95% CI: −41.80 to 34.94, *p* = 0.86, *I*^2^ = 78%). Based on the intervention program, neither the synbiotics group (MD = −4.03 mg/dL, 95% CI: −11.13 to 3.08, *p* = 0.27, *I*^2^ = 0) nor the probiotics group (MD = −11.79 mg/dL, 95% CI: −28.10 to 4.52, *p* = 0.16, *I*^2^ = 69%) showed a significant effect on TG. In conclusion, the heterogeneity of TG appears to be associated with participant region, mean age, diabetes comorbidity status, treatment duration, and intervention program.

LDL-C: Regarding regional differences, Asian participants significantly reduced LDL-C levels (MD = −14.48 mg/dL, 95% CI: −20.76 to −8.21, *p* < 0.00001, *I*^2^ = 50%), whereas European (MD = −0.03 mg/dL, 95% CI: −15.26 to 15.2, *p* = 1.00, *I*^2^ = 72%) and South American participants (MD = 8.05 mg/dL, 95% CI: −5.69 to 21.79, *p* = 0.25, *I*^2^ = 68%) showed no significant effect. In terms of mean age, the <50 years intervention group significantly decreased LDL-C levels (MD = −13.97 mg/dL, 95% CI: −20.25 to −7.69, *p* < 0.0001, *I*^2^ = 48%), while the ≥50 years group showed no significant effect (MD = 4.68 mg/dL, 95% CI: −4.42 to 13.78, *p* = 0.31, *I*^2^ = 75%). Concerning diabetes, neither the non-excluded group (MD = −6.67 mg/dL, 95% CI: −14.29 to 0.95, *p* = 0.09, *I*^2^ = 81%) nor the diabetes-excluded group (MD = −6.18 mg/dL, 95% CI: −14.78 to 2.43, *p* = 0.16, *I*^2^ = 0) showed a significant effect. Regarding treatment duration, the <24 weeks group significantly reduced LDL-C levels (MD = −7.92 mg/dL, 95% CI: −15.07 to −0.77, *p* = 0.03, *I*^2^ = 75%), whereas the ≥24 weeks group showed no significant effect (MD = 0.41 mg/dL, 95% CI: −5.16 to 5.98, *p* = 0.89, *I*^2^ = 0). In terms of the intervention program, the probiotics group significantly reduced LDL-C levels (MD = −6.27 mg/dL, 95% CI: −11.07 to −1.47, *p* = 0.01, *I*^2^ = 27%), whereas the synbiotics group showed no significant effect (MD = −4.61 mg/dL, 95% CI: −18.33 to 9.11, *p* = 0.51, *I*^2^ = 88%). In summary, the heterogeneity of LDL-C appears to be related to participant region, mean age, whether diabetes was excluded, treatment duration, and intervention program.

FPG: Regarding regional differences, both the Asian group (MD = −3.33 mg/dL, 95% CI: −5.35 to −1.30, *p* = 0.001, *I*^2^ = 36%) and the South American group (MD = −10.29 mg/dL, 95% CI: −14.30 to −6.29, *p* < 0.00001, *I*^2^ = 0) showed a significant reduction in FPG levels, whereas no significant effect was observed in the European group (MD = −2.80 mg/dL, 95% CI: −7.58 to 1.98, *p* = 0.25, *I*^2^ = 0). In terms of mean age, the group aged <50 years exhibited a significant decrease in FPG (MD = −3.37 mg/dL, 95% CI: −4.74 to −1.99, *p* < 0.00001, *I*^2^ = 0), while no significant effect was noted in the group aged ≥50 years (MD = −4.26 mg/dL, 95% CI: −11.40 to 2.87, *p* = 0.24, *I*^2^ = 64%). Concerning diabetes, the non-excluded group significantly reduced FPG levels (MD = −4.54 mg/dL, 95% CI: −6.70 to −2.37, *p* < 0.0001, *I*^2^ = 41%), whereas the diabetes-excluded group showed no significant effect (MD = −1.57 mg/dL, 95% CI: −6.30 to 3.17, *p* = 0.54, *I*^2^ = 28%). Regarding treatment duration, interventions lasting <24 weeks showed a significant reduction (MD = −3.47 mg/dL, 95% CI: −4.82 to −2.11, *p* < 0.00001, *I*^2^ = 2%), while the ≥24 weeks group demonstrated no significant effect (MD = −5.51 mg/dL, 95% CI: −15.64 to 4.61, *p* = 0.29, *I*^2^ = 63%). Based on the intervention program, both the synbiotics group (MD = −3.25 mg/dL, 95% CI: −4.73 to −1.77, *p* < 0.00001, *I*^2^ = 0) and the probiotics group (MD = −5.39 mg/dL, 95% CI: −9.00 to −1.78, *p* = 0.003, *I*^2^ = 50%) significantly reduced FPG levels. In summary, the heterogeneity of FPG appears to be associated with participant region, mean age, diabetes comorbidity status, treatment duration, and intervention program.

Insulin: In terms of regional differences, the Asian group significantly reduced insulin levels (MD = −2.73 U/L, 95% CI: −5.43 to −0.03, *p* = 0.047, *I*^2^ = 70%), whereas the South American group significantly increased insulin levels (MD = 1.82 U/L, 95% CI: 0.32 to 3.31, *p* = 0.02, *I*^2^ = 0), and the European group showed no significant effect (MD = −0.44 U/L, 95% CI: −3.09 to 2.20, *p* = 0.74, *I*^2^ = 0). Regarding mean age, insulin levels significantly increased in the ≥50 years group (MD = 1.39 U/L, 95% CI: 0.04 to 2.74, *p* = 0.04, *I*^2^ = 0), while they significantly decreased in the <50 years group (MD = −2.27 U/L, 95% CI: −4.53 to −0.02, *p* = 0.049, *I*^2^ = 60%). Concerning diabetes, the diabetes-excluded group showed a significant reduction in insulin levels (MD = −3.27 U/L, 95% CI: −5.72 to −0.83, *p* = 0.009, *I*^2^ = 0), while the non-excluded group demonstrated no significant effect (MD = −0.26 U/L, 95% CI: −1.97 to 1.45, *p* = 0.76, *I*^2^ = 58%). Based on treatment duration, insulin levels significantly increased in the ≥24 weeks intervention group (MD = 1.82 U/L, 95% CI: 0.32 to 3.31, *p* = 0.02, *I*^2^ = 0), whereas they significantly decreased in the <24 weeks intervention group (MD = −1.81 U/L, 95% CI: −3.59 to −0.03, *p* = 0.047, *I*^2^ = 50%). Regarding the intervention program, neither the synbiotics group (MD = −0.81 U/L, 95% CI: −2.20 to 0.58, *p* = 0.25, *I*^2^ = 29%) nor the probiotics group (MD = −1.88 U/L, 95% CI: −5.78 to 2.02, *p* = 0.35, *I*^2^ = 73%) showed a significant overall effect. In conclusion, the heterogeneity of insulin appears to be associated with participant region, mean age, the exclusion of diabetes, intervention duration, and intervention program.

HOMA-IR: Regarding regional differences, no significant effects were observed in the Asian group (MD = −0.67, 95% CI: −1.38 to 0.04, *p* = 0.07, *I*^2^ = 64%), the European group (MD = −0.69, 95% CI: −1.57 to 0.19, *p* = 0.13, *I*^2^ = 0), or the South American group (MD = 0.37, 95% CI: −0.23 to 0.97, *p* = 0.23, *I*^2^ = 0). In terms of mean age, neither the group aged ≥50 years (MD = −0.21, 95% CI: −1.30 to 0.88, *p* = 0.71, *I*^2^ = 60%) nor the group aged <50 years (MD = −0.59, 95% CI: −1.22 to 0.03, *p* = 0.06, *I*^2^ = 53%) showed a significant effect. Concerning diabetes, the diabetes-excluded group significantly reduced HOMA-IR levels (MD = −0.81, 95% CI: −1.61 to −0.01, *p* = 0.047, *I*^2^ = 3%), whereas the non-excluded group demonstrated no significant effect (MD = −0.28, 95% CI: −0.79 to 0.24, *p* = 0.29, *I*^2^ = 52%). Regarding treatment duration, the <24 weeks group exhibited a significant reduction in HOMA-IR (MD = −0.25, 95% CI: −0.49 to −0.02, *p* = 0.03, *I*^2^ = 49%), while the ≥24 weeks group showed no significant effect (MD = 0.37, 95% CI: −0.23 to 0.97, *p* = 0.23, *I*^2^ = 0). Based on the intervention program, no significant effects were observed in either the synbiotics group (MD = −0.23, 95% CI: −0.73 to 0.26, *p* = 0.36, *I*^2^ = 42%) or the probiotics group (MD = −0.63, 95% CI: −1.50 to 0.23, *p* = 0.15, *I*^2^ = 59%). In conclusion, the heterogeneity of HOMA-IR appears to be associated with participant region, intervention duration, whether diabetes was excluded, and intervention program, but it is not related to mean age.

TNF-α: Regarding regional differences, the Asian intervention group demonstrated a significant reduction in TNF-α levels (SMD = −0.84, 95% CI: −1.12 to −0.56, *p* < 0.00001, *I*^2^ = 16%), whereas both the European group (SMD = 0.56, 95% CI: −0.19 to 1.32, *p* = 0.15, *I*^2^ = 0) and the South American group (SMD = −0.10, 95% CI: −0.67 to 0.47, *p* = 0.73, *I*^2^ = 0) showed no significant effects. Concerning mean age, the <50 years group exhibited significantly lower TNF-α levels (SMD = −0.66, 95% CI: −1.15 to −0.17, *p* = 0.008, *I*^2^ = 71%), whereas no significant effect was observed in the ≥50 years group (SMD = −0.10, 95% CI: −0.67 to 0.47, *p* = 0.73, *I*^2^ = 0). In terms of diabetes comorbidity, a significant reduction in TNF-α levels was only observed in the diabetes-excluded group (MD = −1.03, 95% CI: −1.39 to −0.67, *p* < 0.00001, *I*^2^ = 6%), whereas the non-excluded group showed no significant effect (MD = −0.23, 95% CI: −0.73 to 0.26, *p* = 0.35, *I*^2^ = 65%). Regarding treatment duration, interventions lasting <24 weeks significantly reduced TNF-α levels (MD = −0.63, 95% CI: −1.11 to −0.15, *p* = 0.009, *I*^2^ = 70%), while interventions lasting ≥24 weeks showed no significant effect (MD = −0.51, 95% CI: −1.40 to 0.37, *p* = 0.26, *I*^2^ = 71%). Regarding the intervention program, the synbiotics group significantly reduced TNF-α levels (SMD = −0.91, 95% CI: −1.29 to −0.53, *p* < 0.00001, *I*^2^ = 35%), whereas the probiotics group showed no significant effect (SMD = −0.26, 95% CI: −0.84 to 0.32, *p* = 0.38, *I*^2^ = 67%). In summary, the heterogeneity of TNF-α appears to be associated with participant region, mean age, whether diabetes was excluded, and intervention program, but it is not associated with treatment duration.

IL-6: Regarding regional differences, no significant effects were observed in the Asian group (SMD = −0.62, 95% CI: −1.30 to 0.06, *p* = 0.07, *I*^2^ = 80%) or the European group (SMD = 0.12, 95% CI: −0.62 to 0.87, *p* = 0.74, *I*^2^ = 0). In terms of diabetes comorbidity, neither the diabetes-excluded group (SMD = −0.14, 95% CI: −0.66 to 0.37, *p* = 0.59, *I*^2^ = 13%) nor the non-excluded group (MD = −0.67, 95% CI: −1.55 to 0.20, *p* = 0.13, *I*^2^ = 84%) exhibited any significant effects. Regarding treatment duration, neither the <24 weeks intervention group (SMD = −0.48, 95% CI: −1.25 to 0.28, *p* = 0.22, *I*^2^ = 84%) nor the ≥24 weeks intervention group (SMD = −0.44, 95% CI: −1.17 to 0.29, *p* = 0.24, *I*^2^ = 0) showed a significant effect. Based on the intervention program, neither the synbiotics group (SMD = −0.68, 95% CI: −2.15 to 0.80, *p* = 0.37, *I*^2^ = 93%) nor the probiotics group (SMD = −0.35, 95% CI: −0.78 to 0.09, *p* = 0.12, *I*^2^ = 16%) exhibited any significant effects. In conclusion, the heterogeneity of IL-6 appears to be associated with participant region, intervention duration, and whether diabetes was excluded, but it is not related to mean age or intervention program.

### 3.7 Publication bias

**Figure 7:**
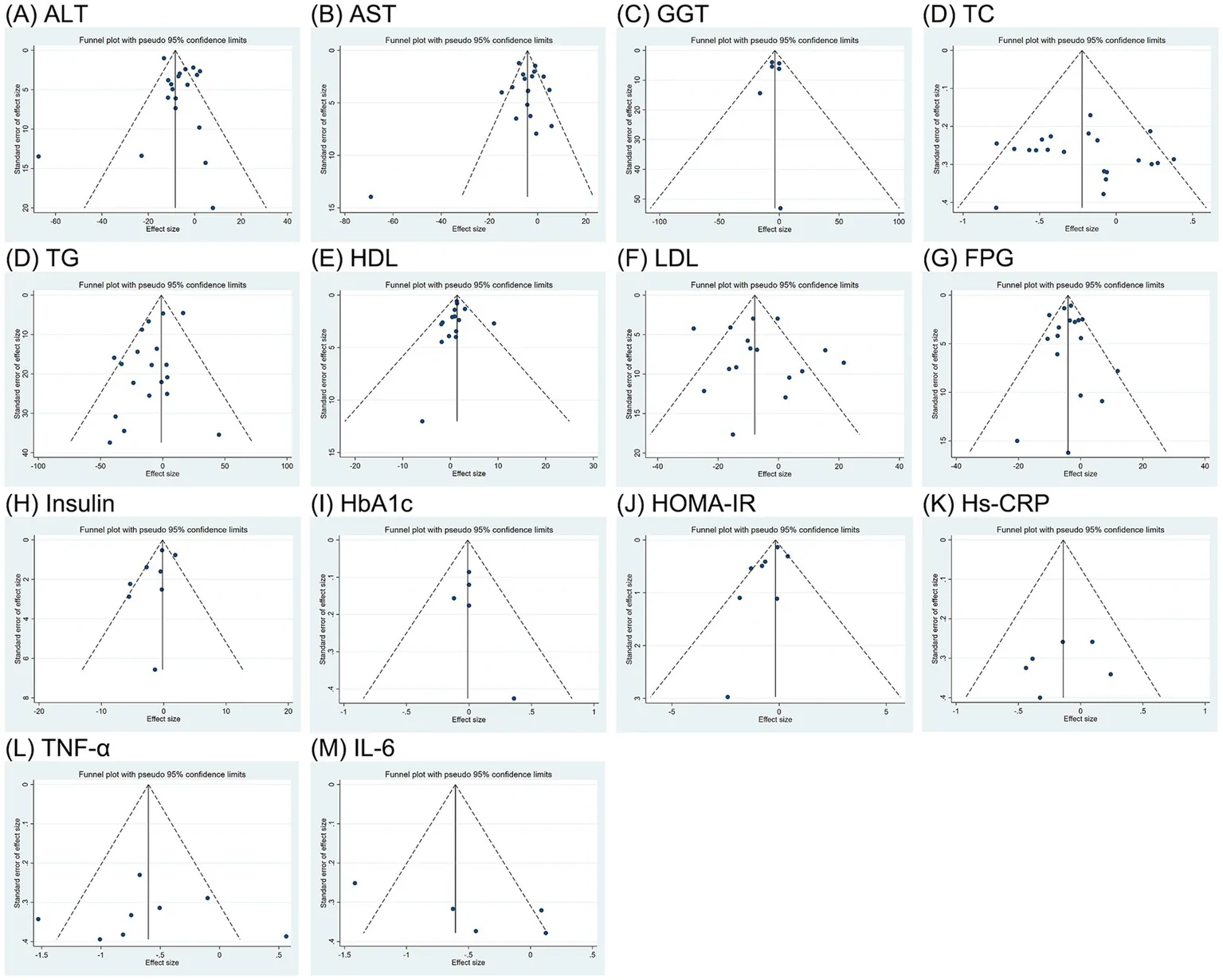
Funnel plots for assessing potential publication bias across clinical outcomes. Funnel plots of standard error by effect size for various outcomes including liver enzymes, lipid and glycemic profiles, and inflammatory markers. Each dot represents an individual study included in the meta-analysis, and the dashed lines indicate the pseudo 95% confidence limits. Symmetry of the scatter plot suggests a low probability of publication bias.

This study employed Egger’s linear regression test to quantitatively assess publication bias for each outcome indicator, as illustrated in Figure 7. The analysis results revealed that among the 14 evaluated indicators, with the exception of TG, the remaining 13 indicators—ALT (*p* = 0.422), AST (*p* = 0.588), GGT (*p* = 0.683), TC (*p* = 0.820), HDL-C (*p* = 0.625), LDL-C (*p* = 0.628), FPG (*p* = 0.806), insulin (*p* = 0.175), HbA1c (*p* = 0.479), HOMA-IR (*p* = 0.111), hs-CRP (*p* = 0.591), TNF-α (*p* = 0.976), and IL-6 (*p* = 0.078)—all yielded *p*-values greater than 0.05 in Egger’s test, indicating the absence of significant publication bias. However, the test result for TG (*p* = 0.034) suggested the potential presence of some publication bias for this particular outcome indicator.

### 3.8 Certainty of evidence

The GRADE framework was used to assess the certainty of evidence for each outcome, with the detailed results presented in Table 4. The certainty of evidence was rated as high for GGT, TC, HDL-C, FPG, HbA1c, and hs-CRP, indicating a high level of confidence in the estimated effects for these outcomes. The certainty of evidence was rated as moderate for AST and TNF-α, suggesting moderate confidence in the pooled estimates, with downgrading mainly related to concerns regarding inconsistency across the included studies. The certainty of evidence for ALT, TG, LDL-C, insulin, HOMA-IR, and IL-6 was rated as low, indicating limited confidence in the pooled estimates and suggesting that further well-designed studies may substantially influence the estimated effects. In particular, the evidence for TG was downgraded because of substantial heterogeneity and potential publication bias (*p* = 0.034). Overall, the low certainty of evidence for several outcomes highlights the need for further large-scale, high-quality randomized controlled trials to confirm the observed effects.

**Table 4:** Certainty of evidence.

Outcome	Risk of bias	Inconsistency	Indirectness	Imprecision	Publication bias	SMD/MD (95%CI)	Certainty of evidence
ALT	None	Very serious	None	None	None	−6.95 (−10.82, −3.07)	Low
AST	None	Serious	None	None	None	−4.38 (−7.19, −1.57)	Moderate
GGT	None	None	None	None	None	−3.67 (−8.27, 0.92)	High
TC	None	None	None	None	None	−0.22 (−0.37, −0.08)	High
TG	None	Serious	None	None	Serious	−8.16 (−17.20, 0.89)	Low
HDL	None	None	None	None	None	1.41 (0.66, 2.16)	High
LDL	None	Very serious	None	None	None	−6.44 (−12.89, 0.02)	Low
FPG	None	None	None	None	None	−4.01 (−6.01, −2.02)	High
Insulin	None	Very serious	None	None	None	−1.09 (−2.79, 0.61)	Low
HbA1C	None	None	None	None	None	−0.01 (−0.13, 0.11)	High
HOMA-IR	None	Very serious	None	None	None	−0.40 (−0.87, 0.06)	Low
hs-CRP	None	None	None	None	None	−0.14 (−0.38, 0.10)	High
TNF-α	None	Serious	None	None	None	−0.60 (−0.99, −0.21)	Moderate
IL-6	None	Very serious	None	None	None	−0.48 (−1.10, 0.14)	Low

SMD, standardized mean difference; MD, mean difference; CI, confidence interval; ALT, alanine aminotransferase; AST, aspartate aminotransferase; GGT, gamma-glutamyltransferase; TC, total cholesterol; TG, triglyceride; HDL-C, high-density lipoprotein cholesterol; LDL-C, low-density lipoprotein cholesterol; FPG, fasting plasma glucose; HbA1c, hemoglobin A1c; HOMA-IR, homeostasis model assessment of insulin resistance; hs-CRP, high-sensitivity C-reactive protein; TNF-α, tumor necrosis factor-alpha; IL-6, interleukin-6.

## 4 Discussion

### 4.1 Study overview

This study comprehensively evaluated the effects of probiotics and synbiotics on liver enzymes, lipid metabolism, glycemic control, and systemic inflammation in patients with MASLD/NAFLD. A recent comprehensive meta-analysis by Xiao et al. (54) also evaluated the effects of probiotics on NAFLD, including 33 studies and 1,940 participants. While their study provided valuable insights, our current review builds upon and distinguishes itself from their work in several key aspects. First, we applied stricter inclusion criteria by limiting eligibility strictly to placebo-controlled trials and excluding studies with incomplete outcome data or a high risk of selective reporting. This minimized bias introduced by varied control regimens and enhanced the reliability of our effect estimates. Second, our literature search was updated to June 2026, incorporating recently published high-quality trials [e.g., Dinavari et al. (37); Won et al. (53)] not captured in prior analyses. Finally, beyond subgroup analyses by geographic region and intervention duration, our updated dataset enabled additional granular stratifications by age (<50 vs. ≥50 years) and baseline diabetic status, yielding more precise and clinically actionable evidence to inform personalized probiotic and synbiotic strategies in MASLD/NAFLD management. In total, 30 RCTs comprising 1,516 participants were included. The results demonstrated that probiotic interventions significantly reduced ALT, AST, TC, FPG, and TNF-α levels, and significantly increased HDL-C levels in patients with MASLD/NAFLD. Overall, probiotics and synbiotics exhibit highly consistent clinical benefits in ameliorating hepatic parenchymal injury and systemic inflammation; however, their modulatory effects on lipid metabolism and insulin resistance are evidently influenced by geographical region, age, and intervention duration.

### 4.2 Effects of probiotics and synbiotics on liver enzymes

Hepatic inflammation and cellular injury are core features of MASLD/NAFLD progression, and changes in ALT and AST levels are closely associated with hepatic steatosis and fibrosis (55, 56). This study confirmed that probiotics and synbiotics can significantly reduce ALT and AST levels in patients with MASLD/NAFLD, which is largely consistent with the findings of previous systematic reviews (57–59). A meta-analysis by Loman et al. (58), which included 25 studies, reported that probiotics and synbiotics significantly decreased ALT (MD = −6.9 U/L, 95% CI: −9.4 to −4.3) and AST (MD = −4.6 U/L, 95% CI: −6.6 to −2.7) levels in NAFLD patients. Similarly, a systematic review by Ma et al. (59) also confirmed that probiotic formulations could significantly reduce ALT (WMD = −23.71, 95% CI: −33.46 to −13.95) and AST (WMD = −19.77, 95% CI: −32.55 to −7.00) in patients with NAFLD. The study by Amini-Salehi et al. (57) also demonstrated significant improvements in ALT and AST. These findings indicate that the effect of probiotics and synbiotics on improving the liver enzyme profile in NAFLD is highly reproducible, establishing hepatoprotection as a core clinical benefit.

However, this study did not find that probiotics and synbiotics could significantly reduce GGT levels. A meta-analysis by Xiao et al. (60) similarly reported that combined probiotic use had no significant effect on GGT levels in patients with NAFLD (*p* = 1.00, *I*^2^ = 97%). In contrast, the majority of earlier meta-analyses, such as those by Loman et al. (58), Ma et al. (59), and Zhou et al. (61), found that probiotics and synbiotics exerted a significant effect on GGT. This discrepancy may be attributed to three main factors. First, recent RCTs have included more cross-ethnic populations, and the high variability in baseline GGT levels might have attenuated the overall effect. Second, there are significant differences in the strain composition and administered dosages of probiotics and synbiotics across the various studies. Finally, from a pathophysiological perspective, ALT and AST are primarily located within hepatocytes, and a decrease in their levels directly reflects the restoration of hepatocyte membrane stability. GGT, on the other hand, is mainly expressed in biliary epithelial cells and is more susceptible to interference from non-parenchymal liver injury factors, such as biliary function and oxidative stress; therefore, its response to gut microbiota interventions may be relatively delayed (62).

### 4.3 Effects of probiotics and synbiotics on lipid metabolism

Dyslipidemia is a key pathological driver of MASLD/NAFLD (63). This study found that although probiotics and synbiotics significantly increased HDL-C levels, the overall pooled effects on TG and LDL-C did not reach statistical significance and exhibited high heterogeneity, whereas TC levels were significantly reduced. Sensitivity analysis indicated that the pooled results for TG were not robust; after excluding the study by Barcelos et al. (29), TG levels showed a significant decrease (MD − 8.27 mg/dL, p = 0.005), suggesting that the true effect on TG may be masked by the clinical heterogeneity of individual studies. Among prior literature, the conclusions of Wang et al. (64) are consistent with our findings, demonstrating no significant effects on TG (SMD = −0.08, 95% CI: −0.42 to 0.25, p = 0.61), TC (SMD = −0.37, 95% CI: −1.07 to 0.32, p = 0.29), and LDL-C (SMD = −0.29, 95% CI: −0.59 to 0.02, p = 0.06). Furthermore, a study by Loman et al. (58) similarly found that probiotic interventions had no significant effect on TC and LDL-C.

Subgroup analysis revealed several noteworthy clinical phenomena. Regarding regional differences, TC, TG, and LDL-C levels significantly decreased in Asian participants post-intervention, whereas TG levels significantly increased in South American participants (with no significant effect observed for TC). The observed regional heterogeneity in treatment effects may be attributable to multiple factors, including, but not limited to, variations in dietary characteristics, host genetic backgrounds, and clinical profiles across regions; however, this remains to be validated in future studies. Regional differences in dietary patterns—such as the intake of fats and dietary fiber—host genetic polymorphisms, and background standard-of-care regimens could potentially modulate the lipid-lowering response to probiotics (65). Nevertheless, as individual-level data on diet or genetics were not extracted in this review, the above interpretations remain untested hypotheses warranting further investigation. In terms of intervention duration, TC, TG, and LDL-C were all significantly reduced in the <24 weeks intervention group, while no significant benefits were observed in the ≥24 weeks long-term intervention group. From a microecological perspective, this may be related to “niche competition” and “colonization resistance.” Long-term supplementation with a single or fixed combination of probiotics and synbiotics, after initially ameliorating dysbiosis, might provoke an adaptive rebound of the host’s indigenous core microbiota. Additionally, when beneficial metabolites such as short-chain fatty acids (SCFAs) reach saturation in the intestinal lumen, they could trigger feedback inhibition mechanisms, leading to diminishing marginal returns from prolonged interventions.

### 4.4 Effects of probiotics and synbiotics on glycemic control and insulin resistance

Insulin resistance (IR) is another core component of metabolic dysfunction in MASLD/NAFLD (66). This study demonstrated that probiotics and synbiotics significantly reduced FPG levels (MD = −4.01 mg/dL, 95% CI: −6.01 to −2.02), but had no significant effect on insulin (MD = −1.09 U/L, 95% CI: −2.79 to 0.61) or HbA1c (MD = −0.01, 95% CI: −0.13 to 0.11). HOMA-IR exhibited a downward trend but did not reach statistical significance (*p* = 0.09). Sensitivity analysis revealed that the heterogeneity for HOMA-IR was primarily associated with the study by Sepideh et al. (51), which may be attributed to the significantly higher probiotic dosage utilized in that trial and an insufficient intervention duration.

Notably, in the subgroup excluding diabetic patients, probiotics and synbiotics significantly lowered insulin and HOMA-IR levels; however, no significant effect was observed in the subgroup that did not exclude diabetic patients. This suggests that for individuals with isolated MASLD/NAFLD who have not yet initiated complex hypoglycemic therapies, the early introduction of probiotics and synbiotics can more effectively improve insulin sensitivity, conferring substantial value for prevention and early intervention. Mechanistically, SCFAs secreted by probiotics can promote GLP-1 secretion by activating free fatty acid receptors (FFAR2/3) (66) and alleviate systemic IR induced by endotoxemia (67).

### 4.5 Effects of probiotics and synbiotics on systemic inflammation

Low-grade chronic inflammation is a pivotal factor in the progression of MASLD to MASH (68). This study confirmed that probiotics and synbiotics significantly reduced TNF-α (SMD = −0.60) level, but had no significant impact on hs-CRP and IL-6. Sensitivity analysis confirmed the robustness of the pooled effect for TNF-α, supporting the reliability of these findings. In previous systematic reviews, multiple RCTs have similarly validated the anti-inflammatory properties of probiotics and synbiotics. For instance, Wang et al. (64) and Yang et al. (69) found that probiotic interventions significantly lowered TNF-α levels in patients with NAFLD. Musazadeh et al. (70) reported that probiotic formulations reduced both hs-CRP and TNF-α levels in patients with NAFLD. Zheng et al. (71) observed that the probiotic intervention group exhibited significantly reduced levels of inflammatory indicators, including TNF-α and IL-6.

However, this study found that the effect of probiotics and synbiotics on hs-CRP was not significant, which is consistent with the meta-analysis by Zhou et al. (61), who also reported no significant improvement in hs-CRP among NAFLD patients following probiotic supplementation. Our results are inconsistent with the findings of Khan et al. (72) and Wang (73). The discrepancies between these study results may stem from various factors, including the specific probiotic strains and the duration of treatment, which could differentially influence systemic inflammation levels in patients with MASLD/NAFLD.

Mechanistically, probiotics and synbiotics can upregulate the expression of intestinal tight junction proteins to fortify the intestinal mucosal barrier, thereby reducing the translocation of intraluminal lipopolysaccharides (LPS) into the portal vein. This fundamentally blocks the overactivation of the TLR4/NF-κB inflammatory signaling pathway in hepatic Kupffer cells (19, 74). However, the lack of significant changes in hs-CRP levels suggests that the anti-inflammatory effects of probiotics and synbiotics may preferentially target specific cytokine networks rather than eliciting a broad acute-phase protein response.

### 4.6 Safety of probiotics and synbiotics in the treatment of MASLD/NAFLD

Among the included studies, only one RCT systematically reported adverse event data (53). This study divided subjects into four groups, receiving either three different probiotic formulations (*n* = 29, 24, and 27) or a placebo (*n* = 23). The results showed that no serious adverse events occurred in any group, and there was no significant difference in the adverse event rate (AER) among the four groups: 3 cases in the LL001 group (AER 10.3%), 4 cases in the LH001 group (AER 16.7%), 6 cases in the PPKID7 group (AER 22.2%), and 2 cases in the placebo group (AER 8.7%). These data provide preliminary evidence for the short-term safety of probiotics and synbiotics in patients with MASLD/NAFLD. However, this conclusion has severe limitations. The study reporting AERs included only 103 patients with a total of 15 adverse events; such a limited sample size may underestimate the true incidence of adverse events. Furthermore, the other included studies did not systematically record adverse events, further limiting the comprehensiveness of the safety assessment. Therefore, there is an urgent need for large-scale, multicenter randomized controlled trials (RCTs) utilizing standardized adverse event monitoring and reporting systems. This will provide more reliable safety evaluations and clarify the benefits and risks of long-term probiotic use in patients with MASLD/NAFLD.

### 4.7 Limitations and future perspectives

This study has several limitations. First, the included studies exhibited high heterogeneity regarding probiotic strains, viable cell dosages, and excipients; empirical administration restricted the evaluation of specific advantageous strains. Second, most studies failed to standardize the baseline lifestyles of the subjects, which could confound the true effects of the microecological interventions. Third, although we attempted to isolate the independent efficacy of probiotics through rigorous eligibility criteria, several included studies employed synbiotics or probiotics combined with vitamins. The synergistic effects of non-probiotic components (e.g., prebiotics, vitamins) could not be completely disentangled through statistical approaches, which may have introduced a certain degree of confounding to the pooled effect estimates. Additionally, reports on the safety and adverse events of probiotic interventions in existing RCTs are relatively scarce. The majority of trials failed to systematically monitor and report the potential risks associated with the long-term supplementation of high-dose live bacterial preparations (e.g., gastrointestinal discomfort or dysbiosis in specific populations), which limits a comprehensive assessment of their safety in clinical practice.

Given the heterogeneity in efficacy and the limitations of safety data, future microecological research on MASLD/NAFLD must transition from clinical observation to precise mechanistic exploration. To address the issue of component confounding, future RCTs should prioritize rigorous single-strain designs to validate the independent effects of specific probiotics. On one hand, it is recommended to employ humanized microbiota-associated (HMA) animal models—transplanting fecal microbiota from human populations with different response profiles into germ-free mice—to replicate metabolic phenotypes and establish causality. On the other hand, integrating network pharmacology with molecular docking technology can facilitate an in-depth investigation of the binding mechanisms between secondary metabolites of specific probiotics and core hepatic metabolic receptors (e.g., FXR, TLR4). Establishing a closed-loop research framework encompassing “clinical data—computational biology—*in vivo* model validation” will provide novel directions for personalized and safe interventions in MASLD/NAFLD.

## 5 Conclusion

In summary, probiotics and synbiotics, as an adjunctive therapy to lifestyle interventions, can significantly and stably ameliorate hepatic parenchymal injury (ALT, AST), systemic inflammation (TNF-α), and specific metabolic parameters (FPG, TC, HDL-C) in patients with MASLD/NAFLD. However, their broader regulatory efficacy on lipid metabolism (e.g., TG, LDL-C) and insulin resistance is significantly influenced by factors such as age, geographical region, and intervention duration. For younger patient populations without diabetes, short- to medium-term interventions (<24 weeks) may yield the optimal metabolic benefits. In clinical practice, homogeneous microecological supplementation strategies should be avoided in favor of exploring precise intervention protocols tailored to the individual metabolic phenotypes of patients.

## Data Availability

The datasets presented in this study can be found in online repositories. The names of the repository/repositories and accession number(s) can be found in the article/Supplementary material.
