# Supplementary material for: Effects of probiotics and synbiotics on liver enzymes, glucose and lipid metabolism, and inflammatory markers in patients with metabolic dysfunction-associated steatotic liver disease: a systematic review and meta-analysis
Source: Front Nutr. 2026 Aug 25;13:1940415. doi: 10.3389/fnut.2026.1940415 (PMC13549844; doi:10.3389/fnut.2026.1940415)
Supplement: Supplementary file 1 [file Table_1.DOCX]

**Supplementary Table S1** The search strategies, filters, and limits for all databases.

| Database | Search strategy | Search dates | Filters | Limits | Number |
| --- | --- | --- | --- | --- | --- |
| PubMed | (((randomized controlled trial[Title/Abstract] OR controlled clinical trial[Title/Abstract] OR randomized[Title/Abstract] OR placebo[Title/Abstract] OR drug therapy[Title/Abstract] OR randomly[Title/Abstract] OR trial[Title/Abstract] OR groups[Title/Abstract])) AND (Probiotic[Title/Abstract] OR Probiotics[Title/Abstract] OR Synbiotic[Title/Abstract] OR Synbiotics[Title/Abstract] OR Bifidobacterium[Title/Abstract] OR Bifidobacteria[Title/Abstract] OR Bacillus bifida[Title/Abstract] OR Yeast[Title/Abstract] OR Saccharomyces cerevisiae[Title/Abstract] OR Saccharomyces italicus[Title/Abstract] OR Saccharomyces oviformis[Title/Abstract] OR S cerevisiae[Title/Abstract] OR S. cerevisiae[Title/Abstract] OR Saccharomyces uvarum var melibiosus[Title/Abstract] OR Candida robusta[Title/Abstract] OR Saccharomyces capensis[Title/Abstract] OR Lactobacillus acidophilus[Title/Abstract] OR Lactobacillus amylovorus[Title/Abstract] OR Lactobacill[Title/Abstract] OR Lactic acid bacteria[Title/Abstract] OR Clostridium butyricum[Title/Abstract] OR Bacillus[Title/Abstract] OR Natto Bacteria[Title/Abstract] OR Streptococcus thermophiles[Title/Abstract] OR Enterococcus[Title/Abstract])) AND (Non alcoholic Fatty Liver Disease[Title/Abstract] OR Nonalcoholic Fatty Liver[Title/Abstract] OR Nonalcoholic Fatty Livers[Title/Abstract] OR NAFLD[Title/Abstract] OR Nonalcoholic Fatty Liver Disease[Title/Abstract] OR Nonalcoholic Steatohepatitis[Title/Abstract] OR Nonalcoholic Steatohepatitides[Title/Abstract] OR Metabolic associated fatty liver disease[Title/Abstract] OR MAFLD[Title/Abstract] OR Metabolic dysfunction associated fatty liver disease[Title/Abstract] OR Fatty Liver[Title/Abstract] OR Steatohepatitis[Title/Abstract] OR Steatohepatitides[Title/Abstract] OR Steatosis of Liver[Title/Abstract] OR Visceral Steatosis[Title/Abstract] OR Visceral Steatoses[Title/Abstract] OR Liver Steatosis[Title/Abstract] OR Liver Steatoses[Title/Abstract]) | June 9, 2026 | None | None | 391 |
| Embase | ('probiotic'/exp OR 'synbiotic agent'/exp OR 'Bifidobacterium'/exp OR 'Saccharomyces cerevisiae'/exp OR 'Lactobacillus'/exp OR 'Lactobacillus acidophilus'/exp OR 'Streptococcus thermophilus'/exp OR 'Bacillus'/exp OR 'Enterococcus'/exp OR 'Clostridium butyricum'/exp OR probiotic*:ti,ab,kw OR synbiotic*:ti,ab,kw OR bifidobacterium:ti,ab,kw OR 'lactic acid bacteria':ti,ab,kw OR lactobacill*:ti,ab,kw OR 'clostridium butyricum':ti,ab,kw OR bacillus:ti,ab,kw) AND ('nonalcoholic fatty liver'/exp OR 'nonalcoholic fatty liver':ti,ab,kw OR 'non-alcoholic fatty liver':ti,ab,kw OR naftd:ti,ab,kw OR 'nonalcoholic steatohepatitis':ti,ab,kw OR nash:ti,ab,kw OR 'fatty liver':ti,ab,kw) AND ('randomized controlled trial'/exp OR 'crossover procedure'/de OR 'double-blind procedure'/de OR 'single-blind procedure'/de OR random*:ti,ab,kw OR factorial*:ti,ab,kw OR crossover*:ti,ab,kw OR placebo*:ti,ab,kw OR ((singl* OR doubl* OR trebl* OR tripl*) NEAR/3 (blind* OR mask*)):ti,ab,kw OR 'clinical trial':ti,ab,kw OR 'controlled trial':ti,ab,kw) | June 9, 2026 | None | None | 536 |
| Cochrane Library | (Probiotic OR Probiotics OR Synbiotic OR Synbiotics OR Bifidobacterium OR Bifidobacteria OR Bacillus bifida OR Yeast OR Saccharomyces cerevisiae OR Saccharomyces italicus OR Saccharomyces oviformis OR S cerevisiae OR S. cerevisiae OR Saccharomyces uvarum var melibiosus OR Candida robusta OR Saccharomyces capensis OR Lactobacillus acidophilus OR Lactobacillus amylovorus OR Lactobacill OR Lactic acid bacteria OR Clostridium butyricum OR Bacillus OR Natto Bacteria OR Streptococcus thermophiles OR EnterococcusProbiotic OR Probiotics OR Synbiotic OR Synbiotics OR Bifidobacterium OR Bifidobacteria OR Bacillus bifida OR Yeast OR Saccharomyces cerevisiae OR Saccharomyces italicus OR Saccharomyces oviformis OR S cerevisiae OR S. cerevisiae OR Saccharomyces uvarum var melibiosus OR Candida robusta OR Saccharomyces capensis OR Lactobacillus acidophilus OR Lactobacillus amylovorus OR Lactobacill OR Lactic acid bacteria OR Clostridium butyricum OR Bacillus OR Natto Bacteria OR Streptococcus thermophiles OR Enterococcus):ti,ab,kw AND (Non alcoholic Fatty Liver Disease OR Nonalcoholic Fatty Liver OR Nonalcoholic Fatty Livers OR NAFLD OR Nonalcoholic Fatty Liver Disease OR Nonalcoholic Steatohepatitis OR Nonalcoholic Steatohepatitides OR Metabolic associated fatty liver disease OR MAFLD OR Metabolic dysfunction associated fatty liver disease OR Fatty Liver OR Steatohepatitis OR Steatohepatitides OR Steatosis of Liver OR Visceral Steatosis OR Visceral Steatoses OR Liver Steatosis OR Liver Steatoses):ti,ab,kw AND (randomized controlled trial OR controlled clinical trial OR randomized OR placebo OR drug therapy OR randomly OR trial OR groups):ti,ab,kw | June 9, 2026 | None | None | 341 |
| Web of Science | TS=(Probiotic* OR Probiotics* OR Synbiotic* OR Bifidobacterium OR Bifidobacteria OR "Bacillus bifida" OR Yeast OR "Saccharomyces cerevisiae" OR "Saccharomyces italicus" OR "Saccharomyces oviformis" OR "S cerevisiae" OR "Saccharomyces uvarum var melibiosus" OR "Candida robusta" OR "Saccharomyces capensis" OR "Lactobacillus amylovorus" OR "Lactobacillus acidophilus" OR Lactobacill* OR "Lactic acid bacteria" OR "Clostridium butyricum" OR Bacillus OR "Streptococcus thermophiles" OR "Natto Bacteria" OR Enterococcus) AND TS=("Non-alcoholic Fatty Liver Disease" OR NAFLD OR "Nonalcoholic Fatty Liver Disease" OR "Nonalcoholic Steatohepatitis" OR"Nonalcoholic Steatohepatitides" OR "Nonalcoholic Fatty Liver" OR NASH OR "fatty liver" OR "hepatic steatosis" OR "Metabolic associated fatty liver disease" OR "Metabolic dysfunction associated fatty liver disease" OR MAFLD OR "Steatohepatitis" OR "Steatosis of Liver" OR "Visceral Steatosis" OR "Visceral Steatoses" OR "Liver Steatosis" OR "Liver Steatoses") AND TS=(randomized OR randomly OR "randomised controlled trial" OR "controlled clinical trial" OR placebo OR trial OR "clinical study" OR "drug therapy" OR "clinical observation" ) | June 9, 2026 | None | None | 588 |
